# Skin-to-blood pH shift triggers metabolome and proteome global remodelling in *Staphylococcus epidermidis*

**DOI:** 10.3389/fmicb.2022.1000737

**Published:** 2022-09-28

**Authors:** Luis Gafeira Gonçalves, Susana Santos, Laidson Paes Gomes, Jean Armengaud, Maria Miragaia, Ana Varela Coelho

**Affiliations:** ^1^Laboratory of Proteomics of Non-Model Organisms, Instituto de Tecnologia Química e Biológica António Xavier, Universidade Nova de Lisboa, Oeiras, Portugal; ^2^Département Médicaments et Technologies pour la Santé, SPI, Université Paris-Saclay, CEA, INRAE, Bagnols-sur-Cèze, France; ^3^Laboratory of Bacterial Evolution and Molecular Epidemiology, Instituto de Tecnologia Química e Biológica António Xavier, Universidade Nova de Lisboa, Oeiras, Portugal

**Keywords:** *Staphylococcus epidermidis*, proteomics, metabolomics, infection, colonization, pH adaptation

## Abstract

*Staphylococcus epidermidis* is one of the most common bacteria of the human skin microbiota. Despite its role as a commensal, *S. epidermidis* has emerged as an opportunistic pathogen, associated with 80% of medical devices related infections. Moreover, these bacteria are extremely difficult to treat due to their ability to form biofilms and accumulate resistance to almost all classes of antimicrobials. Thus new preventive and therapeutic strategies are urgently needed. However, the molecular mechanisms associated with *S. epidermidis* colonisation and disease are still poorly understood. A deeper understanding of the metabolic and cellular processes associated with response to environmental factors characteristic of SE ecological niches in health and disease might provide new clues on colonisation and disease processes. Here we studied the impact of pH conditions, mimicking the skin pH (5.5) and blood pH (7.4), in a *S. epidermidis* commensal strain by means of next-generation proteomics and ^1^H NMR-based metabolomics. Moreover, we evaluated the metabolic changes occurring during a sudden pH change, simulating the skin barrier break produced by a catheter. We found that exposure of *S. epidermidis* to skin pH induced oxidative phosphorylation and biosynthesis of peptidoglycan, lipoteichoic acids and betaine. In contrast, at blood pH, the bacterial assimilation of monosaccharides and its oxidation by glycolysis and fermentation was promoted. Additionally, several proteins related to virulence and immune evasion, namely extracellular proteases and membrane iron transporters were more abundant at blood pH. In the situation of an abrupt skin-to-blood pH shift we observed the decrease in the osmolyte betaine and changes in the levels of several metabolites and proteins involved in cellular redoxl homeostasis. Our results suggest that at the skin pH *S. epidermidis* cells are metabolically more active and adhesion is promoted, while at blood pH, metabolism is tuned down and cells have a more virulent profile. pH increase during commensal-to-pathogen conversion appears to be a critical environmental signal to the remodelling of the *S. epidermidis* metabolism toward a more pathogenic state. Targeting *S. epidermidis* proteins induced by pH 7.4 and promoting the acidification of the medical device surface or surrounding environment might be new strategies to treat and prevent *S. epidermidis* infections.

## Introduction

*Staphylococcus epidermidis* is one of the most important skin commensals (Becker et al., 2014). However, it is also a major opportunistic pathogen (Gill et al., 2005; Brown et al., 2012) associated with medical devices-related infections in immunocompromised patients (Conlan et al., 2012; Sabaté Brescó et al., 2017). These infections result from the disruption of the skin barrier by incision during surgery or introduction of catheters which allows *S. epidermidis* access to the bloodstream (Kleinschmidt et al., 2015; Lee and Anjum, 2022) and followed by production of biofilms on medical device’s surface. During skin to blood transition, *S. epidermidis* must cope with drastic environmental changes. On the skin, *S. epidermidis* is subjected to UV radiation, low nutrient availability, dryness, salinity fluctuations, low temperatures and a low pH range (pH 4.5–7; average pH 5.5) (Lambers et al., 2006; Schmid-Wendtner and Korting, 2006; Kammeyer and Luiten, 2015; Espadinha et al., 2019). In contrast, in the blood, *S. epidermidis* faces a completely different environment: richer in nutrients, with higher temperature and more alkaline (pH 7.4). Moreover, in the blood *S. epidermidis* has to deal with the presence of pro-inflammatory molecules and reactive oxygen species (ROS) generated by immune cells (Akira et al., 2006; Weiss and Schaible, 2015; Espadinha et al., 2019) and the mechanical stress imposed by the blood flow that was previously shown to interfere with bacterial growth (Viela et al., 2019; Steinert et al., 2020). Despite these obvious differences between commensal and pathogenic environments, the mechanisms underlying *S. epidermidis* adaptation to blood conditions are yet poorly understood (Espadinha et al., 2019), in part due to the complexity of the skin and blood environment. Skin is a low acidic environment that potentially has antimicrobial activity since the undissociated forms of weak acids pass freely through the bacterial cell membrane. Since the cytoplasmic pH is generally higher than that of the growth medium, the weak acid dissociates leading to acidification of the cytoplasm. This effect can induce structural damage to the cell membrane and macromolecules, such as DNA and proteins. Bacterial survival strategies to deal with the environmental acidic stress include the combination of constitutive and inducible strategies that result in the removal of protons, namely transmembrane proton motive force, alkalisation of the external environment, changes in the composition of the cell envelope, production of general shock proteins and chaperones, expression of transcriptional regulators, and responses to changes in cell density, in particular when part of a biofilm (Cotter and Hill, 2003). At blood pH, non-CC2 strains of clonal lineage B produce a significant lower amount of biofilm (Espadinha et al., 2019).

In the recent years, proteomic and metabolomic approaches to study bacterial physiology and infection has gain momentum (Bonar et al., 2015, 2018; Chen et al., 2017; Jean Beltran et al., 2017). In the case of *S. epidermidis*, proteomic and metabolomics was used to study biofilm formation (Carvalhais et al., 2015; Harris et al., 2016; Bottagisio et al., 2019; Gu et al., 2020; Martínez-García et al., 2021), to compare commensal and pathogenic strains (Savijoki et al., 2014; Siljamäki et al., 2014; Águila-Arcos et al., 2015), to study the processes associated to stress response (Zhang et al., 2011) and the adhesion to medical devices (Sadykov et al., 2010; Bürgers et al., 2018; Bottagisio et al., 2020).

To get new insights on the mechanisms of *S. epidermidis* adaptation during infection we analyzed *S. epidermidis* metabolic response to skin and blood pH. A *S. epidermidis* strain belonging to the less pathogenic *S. epidermidis* lineage (B lineage) (Espadinha et al., 2019), was grown at pH 5.5 and 7.4, and LC-MSMS proteomic and NMR metabolomic data were acquired for their intracellular content. Integrated functional analysis of the differentially regulated proteins and metabolites between the two pH conditions identified pathways and cellular processes clearly associated to commensal and pathogenic states, suggesting that a pH shift is a key signal for pathogenicity in this bacterial species.

## Materials and methods

### Ethical statement

*Staphylococcus epidermidis* isolate 19N was obtained from screening of the anterior nares of draftees attending Centro de Formação da Ota (Lisbon, Portugal), which was performed with written informed consent and approval from all the necessary military authorities. The procedure was non-invasive.

### Bacterial strains and growth conditions

The *S. epidermidis* 19N strain was collected from the anterior nares of a healthy person in Portugal in 2001. This strain was previously characterised by whole genome sequencing and belongs to clonal lineage B (Espadinha et al., 2019).

A single colony from a *S. epidermidis* 19N strain culture grown O/N at 37°C (TSA, BactoTM), was used to pre-inoculate Tryptic Soy Broth (TSB) medium with two different pH (5.5 and 7.4) that was incubated overnight at 37°C at 225 rpm. The volume of the culture medium was kept at 36% (90 ml/250 ml) of the flask total volume to ensure a good aeration. Pre-inoculums were adjusted either to pH 5.5 or pH 7.4, with hydrochloric acid.

### Experimental modelling—stress challenge

**FIGURE 1:**
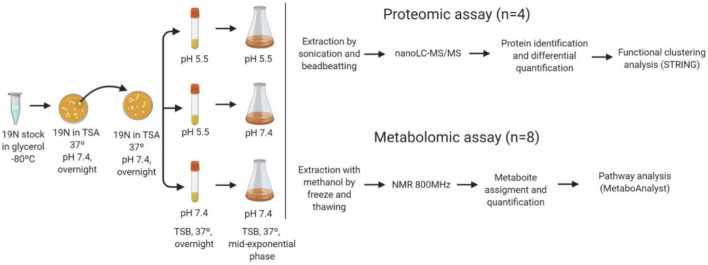
Scheme of the workflow followed. Cells from –80°C glycerol stocks were streaked in Tryptic Soy agar media (TSA) and a single colony was picked to inoculate a new TSA plate. From the plate a single colony was collected to prepare a pre-inoculum in TS broth medium with adjusted pH (pH 5.5 and 7.4) and incubated overnight at 37°C and 225 rpm. The inoculum at pH 5.5 was used for the cultures grown at 5.5 (N55) and 7.4 (N57), and the inoculum at pH 7.4 for the culture at the same pH (N77). The cells were harvested at mid-exponential phase. For proteomics the proteins were extracted from 4 biological replicates/experimental condition and analysed by nanoLC-MS/MS. Identified proteins (MASCOT) were quantified by spectral counts. Differential abundant proteins between experimental conditions were submitted to functional clustering analysis (STRINGdb). Metabolites from eight replicates/experimental condition were extracted, after metabolic quenching and biomass normalisation. Metabolites were assigned in ^1^H-NMR spectra acquired in an 800 MHz spectrometer (ChenomxNMRsuite). Their determined concentrations were used for pathway analysis (MetaboAnalyst).

In this work, three pH transitions from pre-inoculum to inoculum were assayed. *S. epidermidis* pre-inoculums and the growth were performed at TSB medium with pH 7.4, to mimic the blood pH; and pH 5.5, to mimic the skin pH. The pre-inoculum cellular density was adjusted to 0.06 (OD_600_) (aprox.1.5 × 10^8^ CFU/mL) and used to inoculate fresh medium in the three conditions depicted in Figure 1, simulating *S. epidermidis* at skin and blood and a pH shock endured by *S. epidermidis* during the infection process from skin to blood transition. The cell cultures incubated at 37°C with 225 rpm were followed by OD_600_ and recovered at mid-exponential phase for further analysis.

### Proteomic analysis

Protein extraction was done on *S. epidermidis* cells harvested at the mid-exponential phase from 100 mL of cell cultures. Four biological replicates were treated per condition. The cultures were centrifuged at 10,000 *g* for 5 min, and the resulting cell pellets were immediately frozen in liquid nitrogen and stored at –80°C. Cells were resuspended in NuPAGE LDS Sample Buffer 1X (Invitrogen, 100 μL/10 mg of pellet) and subjected to bead-beating as described (Hayoun et al., 2019). The proteins were then treated as detailed previously (Rubiano-Labrador et al., 2014) to obtain tryptic peptides. These were analysed with a Q-Exactive HF high resolution tandem mass spectrometer (ThermoFisher Scientific) incorporating an ultra-high-field Orbitrap analyzer (Klein et al., 2016). Shortly, peptide mixtures were injected and desalted online using a reverse phase precolumn Acclaim PepMap 100 C18 and resolved on a reverse phase column Acclaim PepMap 100 C18 with a flow rate of 200 nl/min with a 90 min gradient. The mobile phase consisted of 0.1% HCOOH (A) and 80% CH_3_CN, 0.1% HCOOH (B) using a gradient programmed from 4 to 25% of mobile phase B for 75 min and 25–40% of mobile phase B for 15 min. Mass spectrometry data-dependent acquisition was performed according to a Top20 strategy consisting in a scan cycle initiated with a full scan of peptide ions in the ultra-high-field Orbitrap analyzer, followed by selection of the precursor, high energy collisional dissociation and MS/MS scans on the 20 most intense precursor ions, selecting threshold intensity of 83,000, potential charge states of 2+ and 3+, and dynamic exclusion of 10 s. Full scan mass spectra were acquired from *m/z* 350 to 1,500 with a resolution of 60,000. Each MS/MS scan was acquired at a resolution of 15,000 with a maximum ion trapping of 60 ms and an *m/z* isolation window of 2.0.

#### Proteome data processing

MS/MS spectra were searched using MASCOT 2.5.1 software (Matrix Science) against the 19N *S. epidermidis* genome for which 2,469 protein sequences have been annotated totalling 720,252 residues (NCBI database SRX7846931). The following parameters were used for MS/MS spectra assignation: full-trypsin specificity, maximum of two missed cleavages, mass tolerances of 5 ppm and 0.02 Da on the parent ion and fragments, respectively, fixed modification of carbamidomethyl cysteine (+57.0215), and oxidized methionine (+15.9949) and deamidated asparagine/glutamine (NQ) (0.9840) as dynamic modifications. Peak lists generated from all spectra of peptides were submitted to MASCOT. Peptides with a score below a *p*-value of 0.05 were assigned to proteins. The proteins were validated when at least two different peptide sequences were detected. The false discovery rate for protein identification was below 1% when applying these rules with the corresponding MASCOT decoy search mode. For each validated protein, spectral count (SC) was used as a proxy of their relative abundances in each condition. SC values for each protein were compared between pH conditions by the calculation of a relative protein enrichment, the T-fold change, as defined by Carvalho after normalisation of the dataset as recommended (Liu et al., 2004; Carvalho et al., 2012). Differentially abundant proteins were selected when their adjusted *p*-value ≤ 0.05 and absolute T-fold above 1.5. The proteins differentially abundant in the 3 conditions were considered for the functional interactions network construction using STRINGdb (v11.0)^1^ based on: co-expression, text-mining, biochemical/genetic data (“experiments”), and previously curated pathway and protein-complex knowledge (“databases”), with *S. epidermidis* RP62A as reference organism and a minimum interaction score of 0.7 (Szklarczyk et al., 2019). For protein functional analysis, BLATSp analysis was performed against *S. epidermidis* database from UniProt (22530 entries in 10/06/2019), considering valid results for which 75% sequence coverage, 35% sequence identity and *p*-value ≤ 0.05 were at least obtained. The mass spectrometry proteomics data have been deposited to the ProteomeXchange Consortium via the PRIDE partner repository (Perez-Riverol et al., 2022) with the dataset identifier PXD034825 and 10.6019/PXD034825.

### Metabolomic analysis

#### Metabolites extraction

Cells were recovered at mid-exponential phase from 100 mL cultures following a protocol adapted from Somerville and Powers (2014). Eight biological replicates of each independent growth condition were obtained. Cells were harvested by centrifugation at 5,000 × *g* for 5 min at 4°C. Cells were washed with 20 mM phosphate buffer pH 7.2–7.4 and centrifuged for 1 min at 13,000 rpm. Cell pellet was suspended in the same buffer with a final OD_600_ of 20 and stored at –80°C for further metabolite extraction. Cells were thawed in a water bath at room temperature and 750 μL of 60% methanol were added and subjected to three freeze-thaw cycles using liquid nitrogen. Extracted samples were centrifuged at 21,000 *g* for 5 min at 4°C. The extraction process on the pellets was repeated twice. The supernatants were kept and stored together at –20°C overnight and dried in a SpeedVac. Dried samples were dissolved in: 750 μL phosphate buffer (33 mM, pH 7.0 in D_2_O with 2 mM of sodium azide) with 0.21 mM of 3-(trimethylsilyl)propionic-2,2,3,3-d_4_ (TSP). The suspensions were centrifuged at 21,000 *g* for 5 min at 4°C and the resulting supernatants were then transferred to 5 mm NMR tubes.

#### NMR metabolite analysis

NMR spectra were acquired on a Bruker Avance II + 800 MHz spectrometer equipped with a 5 mm TXI-Z H/C/N/-D probe. All 1D ^1^H were acquired at 298.15 K and using a *noesygppr1d* pulse program [256 scans; relaxation delay of 4 s; mixing time of 10 ms; spectral width of 16025.641 Hz; size of free induction decay (FID) was 128k points]. Processing of spectra was performed with Bruker TopSpin 3.2. All FID were multiplied by an exponential function, followed by Fourier Transformation. Spectra were manually phased and baseline corrected. Chemical shifts were adjusted according to the TSP chemical shift at 0.00 ppm. For spectral assignment, 2D NMR spectra were acquired for some samples: ^1^H-^1^H TOCSY, ^1^H-^13^C HSQC, and ^1^H *J*-resolved.

1D ^1^H NMR processed spectra were grouped and converted into a matrix (each column was a spectrum; each row was one of the 128k points that makes up the FID). Spectra were aligned and centred according to the chemical shift of TSP at 0.00 ppm. For untargeted analysis, the region of water (4.70--4.95 ppm) was removed. After trimming interfering peaks, only the region between 0.15 and 10.00 ppm was used. All the aforementioned steps were completed using R software environment for statistical computing (v3.6.2) with homemade scripts. For targetted analysis, resonances assignment, metabolites identification and quantification were performed with Chenomx NMR suite 8.12. All the metabolites were confirmed based on two-dimensional NMR spectra. Metabolomic data is available at the NIH Common Fund’s National Metabolomics Data Repository (NMDR) website, the Metabolomics Workbench,^2^ where it has been assigned Project ID ST003341.

### Statistical analysis

The results were evaluated by univariate and multivariate statistical analysis. In the case of proteomic data, a univariate t-Student analysis was performed using bicaudal distribution and homoscedastic samples. For metabolite data, pairwise comparisons were conducted using the Wilcoxon rank-sum test to look for significant differences (*p*-value ≤ 0.05) between the groups of samples. Correction for multiple testing was performed using the Benjamini-Hochberg procedure (Benjamini and Hochberg, 1995). This analysis was performed in the R software environment for statistical computing (v3.6.2). Multivariate statistics included principal component analysis (PCA) and partial least squares discriminant analysis (PLS-DA) for both data sets. A cross-validation method was used to evaluate the goodness of prediction (Q2) value of the resulting models, with a permutation analysis to further confirm the validity of the PLS-DA model. To understand which metabolites were contributing the most to the separation observed by PLS-DA, a variable importance in projection (VIP) analysis was conducted using the loadings derived from the PLS-DA. For the proteomic data, Hierarchical Cluster analysis was performed with MetaboAnalyst 4.0^3^ (Chong et al., 2019). Pathway analysis was done to identify the putative pathways altered between the different conditions based on the metabolites levels using the *Staphylococcus aureus* N315 as the pathway library reference in MetaboAnalyst 4.0 (Xia and Wishart, 2010). Proteins differentially abundant between pH conditions were considered to the network construction using STRING database (Szklarczyk et al., 2019) with (0.9) confidence minimum required interaction score.

## Results

### *Staphylococcus epidermidis* 19N growth conditions and multi-omics comparative strategy

In order to understand the changes occurring in each condition that mimic colonisation and infection the proteome and metabolome of *S. epidermidis* 19N strain were determined at pH 5.5 (hereafter N55), pH 7.4 (hereafter N77) and at pH 7.4 but with a culture initiated with a pre-inoculum grown at pH 5.5 (hereafter N57). This last condition simulated a sharp pH alteration as expected during infection. Figure 1 shows the experimental strategy for obtaining for each experimental condition four and eight biological replicates for proteomic and metabolomic analysis, respectively. Growth curves were monitored (Supplementary Figure 1), showing a significantly higher growth rate for the N57 transition condition (0.978 h^–1^) compared to the N55 (0.817 h^–1^) and N77 (0.705 h^–1^) steady conditions. The growth rate is also significantly different between these two last conditions. Measured media pH values at the middle-exponential phase have dropped 0,3 and 1,0 units, respectively for N55 and N77.Cells were harvested at the mid-exponential phase and subjected to shotgun proteomics and NMR-based metabolomics.

With the proteomic workflow used a total of 771,440 MS/MS spectra were acquired for the 12 analysed samples. A total of 441,537 Peptide-to-Spectrum Matches (PSMs) were interpreted, resulting in a ratio of 57% of assigned MS/MS spectra. Such a ratio is usually obtained in similar conditions when a highly confidently annotated genome is used as a database. The dataset gave confident identification of 20,194 unique peptides and 1,372 proteins that could be quantified. This number is roughly similar for the three experimental conditions: 1347 (N55), 1353 (N57), and 1351 (N77). High reproducibility was observed within the biological replicates of each experimental condition (see Supplementary Table 1, sheet “Supplementary Table 1”). By NMR it was possible to identify and quantify 46 metabolites common among the analysed samples. From 187 resonances present in the ^1^H-NMR spectra, it was possible to assign most of the most intense ones, in a total of 142 (76%).

### Differential proteomic analysis

**FIGURE 2:**
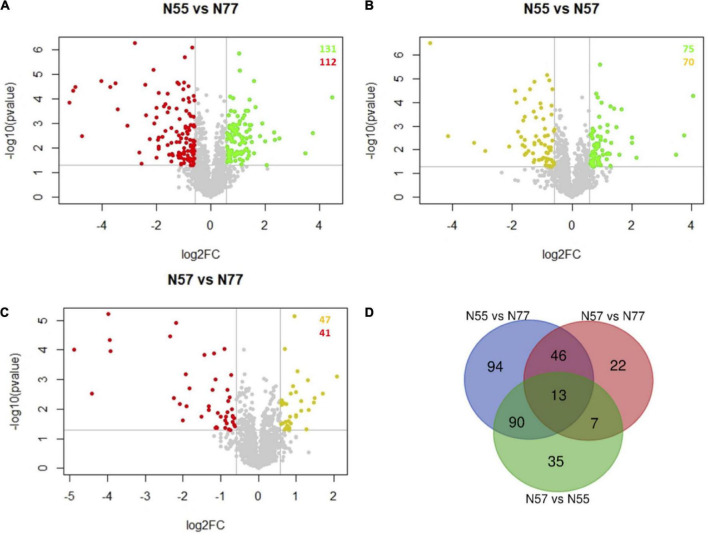
Volcano plots and Venn diagram of protein abundance differences between conditions. **(A–C)** Volcano plots of the proteins differentially abundant between each pair of experimental conditions (FC > 1.5, *p*-value < 0.05). Spots representing proteins with increased abundances in N55, N57 and N77 are coloured in red, yellow and green, respectively. **(A)** N55 vs N77; **(B)** N55 vs N57; and **(C)** N57 vs N77. **(D)** Venn diagram showing the number of proteins that are differentially abundant in the several comparisons of experimental conditions (N55 vs N77, N55 vs N57 and N57 vs N77).

A multivariate statistical analysis showed that the proteomic datasets for the three conditions were clearly distinguished by the PCA test, and no biological replicate should be considered as outlier. Five components from the PCA test explains 81% of the variance among the three experimental conditions (Supplementary Figure 2). The Volcano plots showing the differential abundance of proteins between each pair of experimental conditions (N55 vs. N77, N55 vs. N57, and N57 vs. N77) are represented in Figures 2A–C. N55 (skin pH condition) vs N77 (blood pH condition) showed 243 proteins with significantly different levels, 131 proteins are increased in N55 condition and 112 increased in N77 (Figure 2A). This constitutes the pair with a higher number of differences by opposition to the comparison N57 vs N77 with only 88 protein abundance differences. A Venn diagram (Figure 2D) reveals the intersection of protein groups with differential levels between each experimental comparison. Thirteen proteins were differentially abundant in the three comparisons (Figure 2D). These include secreted proteins (like proteases, signal peptidases and a secretory antigen) and others involved in glycerolipids metabolism, biosynthesis of polyphosphates, betaine and DNA replication. The highest number of proteins with abundance differences specific to a pair of conditions was also observed for N55 vs N77 (94 proteins) and the lowest for N57 vs. N77 (22 proteins), suggesting, respectively, a higher and a lower change of the biological and metabolomic processes associated to these two pairs.

**FIGURE 3:**
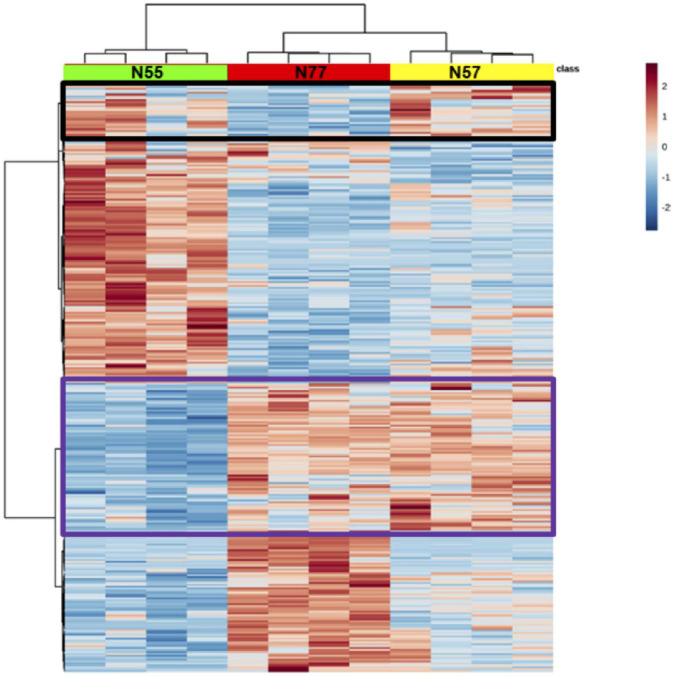
Hierarchical clustering of differentially abundant proteins. In green, red and yellow are identified the columns for the replicates of each experimental condition, respectively N55, N77, and N57. In the heatmap, red and blue correspond to higher and lower abundant proteins, respectively. The tree in the x axis represents the similarity among experimental conditions regarding abundance of proteins and in the y axis the proteome similarities. Proteins having increased levels in common between conditions N55 and N57 and conditions N57 and N77 are evidenced in black and purple rectangles, respectively.

Hierarchical clustering of differentially abundant proteins confirmed a clear separation among the three experimental conditions (Figure 3). Few proteins increased in N55 are also increased in N57, around half of the proteins increased in N77 are also increased in N57, suggesting that the biological and metabolic processes associated to N77 are closer to those prevailing in N57 and that this condition appears as a transition between the two pH environments, skin, and blood.

#### Proteomic analysis at skin vs blood environmental pH

The proteins differentially detected (Supplementary Table 1, sheet “Supplementary Table 1_N55vsN77”) were analysed in terms of STRINGdb interaction networks to find potentially modulated global metabolic pathways. A total of 243 differential proteins were detected between blood and skin pH.

**FIGURE 4:**
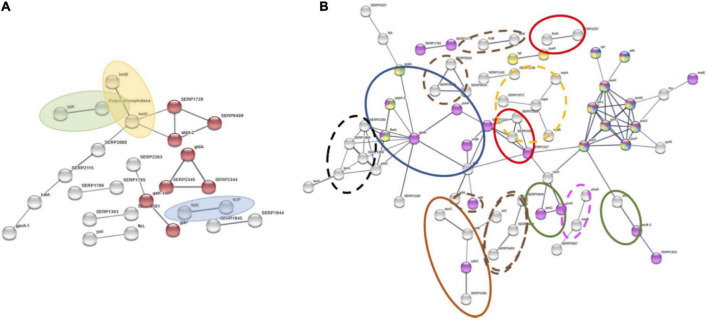
STRING network of differentially abundant proteins between N55 and N77 conditions. Proteins increased **(A)** and decreased **(B)** in condition N55 when compared with N77. **(A)** Proteins associated with glycerolipid metabolism are represented as red nodes. Blue, yellow and green shadowed ellipsis highlight proteins involved in folate and betaine biosynthesis and phosphate metabolism. **(B)** Proteins associated to purine and nucleotide metabolism are represented as red, purple, green and yellow nodes, while those associated with the biosynthesis of secondary metabolites are represented as pink nodes, including proteins involved in glycolysis/gluconeogenesis/pyruvate metabolism, oxidative phosphorylation, acetoin catabolism arginine and proline metabolism circled by blue, orange, red and green open ellipsis. The other clusters highlighted with dashed black, brown, yellow and magenta ellipsis are the phosphoenolpyruvate:sugar phosphotransferase system (PTS), membrane transporters, extracellular proteases and the SaeRS two-component signal transduction system (TCS).

The network for the 112 proteins whose abundance increased in N55 exhibits 27 nodes (PPI enrichment *p*-value = 5.69e-08) of which 8 are related with the glycerolipid metabolism (Figure 4A). Furthermore, it highlights oxidative phosphorylation, metabolism of phosphate and biosynthesis of betaine and of folate. Betaine is a modified amino acid that works as an osmoprotectant and as a methyl donor, while folate is an enzyme cofactor also involved in methylation reactions relevant in the biosynthesis of purines. The transport of drugs and cell division are also relevant in N55. In particular *N*-acetylmuramoyl-L-alanine amidase, involved in the cleavage of the amide bond between *N*-acetylmuramoyl and L-amino acids in bacterial cell walls, which is the most differentially increased protein.

On the other hand, for the 131 proteins identified as increased in N77 a 69 nodes STRING network was established (PPI enrichment *p*-value = 5.55e-16) (Figure 4B). In this case the most represented metabolic pathways were the purine/nucleotide metabolism, glycolysis/pyruvate metabolism, oxidative phosphorylation, butanoate metabolism, and arginine and proline metabolism. Other STRING clusters are associated with the phosphoenolpyruvate:sugar phosphotransferase system (PTS); hemin, iron, and manganese and other membrane transporters; extracellular proteases, t-RNA modification and; the SaeRS two-component signal transduction system (TCS), which influences virulence and biofilm formation in *S. aureus* (Lou et al., 2011). The proteins with a higher abundance increment in N77 are glyceraldehyde-3-phosphate dehydrogenase (glycolysis/gluconeogenesis) and two extracellular proteases, staphopain A and a glutamyl endopeptidase. In particular, staphopain A is a cysteine protease that plays an important role in the inhibition of host innate immune response. In *S. aureus* this protein was shown to cleave host elastins found in connective tissues, facilitating invasion of host cells and to hydrolyse the chemokine receptor CXCR2 on leukocytes, which blocks neutrophil activation and chemotaxis (Potempa et al., 1988; Kantyka et al., 2013). On the other hand, the glutamyl endopeptidase (SspA) from *S. epidermidis* was shown to exhibit a significant hydrolytic activity for the carbonyl side of glutamic acid and to show activity toward human fibronectin and type 1 collagen (Ohara-Nemoto et al., 2002).

#### Proteomic comparison for pH shock adaptation vs blood and skin mimicking conditions

When we compared *S. epidermidis* 19N cultures that faced a sudden pH shock (condition N57) with cultures adapted to pH 5.5 (N55) or 7.4 (N77) (i.e., N57 vs. N55 and N57 vs. N77, respectively), the number of proteins differentially abundant is smaller in both cases than for the comparison between N55 vs. N77. This is an expected situation, since in N57 the bacterial cells did not have the required time to adapt completely to the new pH condition (pH 7.4), as occurs in condition N77, when the inoculum pH was maintained and the metabolism had the opportunity to be re-routed showing abundance differences for more proteins.

In the case of N55 vs N57, there are 145 unique proteins with significantly different levels, 75 are increased in N55 (Figure 2B), of which 54 are also increased in N55 when compared with N77. The biosynthesis of betaine and the metabolism of phosphate are still increased in N55, probably being both processes quickly adjusted when 19N starts growing at pH 7.4. However, less proteins are involved the glycerolipid metabolism and the biosynthesis of folate were incremented, suggesting that these metabolic pathways require a longer time to respond to the pH change. Most likely due to the pH shock, other N55 processes had to be deactivated at N57 but not at N77. These include maintenance of redox homeostasis (5 oxidoreductases, SERP2129, SERP1917, SERP2165, SERP0244 and nfrA, were significantly reduced at N57), betaine membrane transport (SERP0246 and SERP2179), insertion of integral membrane proteins (SERP1356) and adjustment of the cell-wall protein composition (SERP2279, sdrH, gtf1). Interestingly, an endoribonuclease from the type II toxin-antitoxin system, MasF, only decreased at N57 relative to N55, was described as an inducer of bacterial dormancy (Bezrukov et al., 2021). Several processes were triggered at N57, most of them also incremented at N77 when compared to N55, such as metabolism of acetoin, PTS, pyruvate fermentation, transmembrane transport (including of hemin), and biosynthesis of purines, TCS, extracellular proteases.

Comparing conditions N57 vs N77, a total of 88 unique differentially abundant proteins were quantified, among them 47 proteins are increased in N77 (Figure 2C), of which 32 are also increased in N77 when compared with N55. From the 41 proteins increased in N57, 14 are not increased in N55 vs N77, those include four proteins related with the reshape of the cell wall (*pbp1*, *vraR*, *ypfP*, and SERP2383). SERP2383 is also involved in the transport of Zn, which could be associated with another increased protein (Fur) that regulates Zn uptake. String network analysis for conditions N55 vs N57 and N57 vs N77 are provided in Supplementary Figure 3.

### Differential metabolomic analysis

A total of 48 metabolites were confidently identified and quantified for the *S. epidermidis* samples from the ^1^H-NMR spectra (Supplementary Figure 4). Most of the metabolites identified are amino acids (35%), carboxylic acids (16%), nucleotides and their constituents (14.5%), sugars (6%), and cofactors (6%). Univariate analysis of differentially accumulated metabolites between the three experimental conditions is summarised in Table 1.

**TABLE 1:** Identified metabolites from *S epidermidis* 19N strain intracellular extract by ^1^H-NMR with respective levels in each experimental condition.

Metabolites	N55		N57		N77		N55/N77	N55/N57	N57/N77
	Mean (nmol)	SD (nmol)	Mean (nmol)	SD (nmol)	Mean (nmol)	SD (nmol)	Fold change	Fold change	Fold change
Acetate	60.7	9.1	59.0	13.0	134.3	28.6	0.5***	1.0	0.4***
Adenine	11.1	7.0	8.3	4.2	15.4	5.8	0.7	1.3	0.5*
Alanine	44.6	19.3	27.2	13.9	51.4	19.6	0.9	1.6	0.5*
Asparagine	32.7	15.9	74.1	36.7	93.5	16.6	0.4***	0.4*	0.8
Aspartate	142.5	25.0	220.0	41.4	434.3	69.2	0.3***	0.6**	0.5***
Betaine	1326.8	171.8	402.5	237.3	1254.2	149.8	1.1	3.3***	0.3***
Choline	32.0	7.6	4.5	1.4	15.2	1.5	2.1***	7.0***	0.3***
Cystathionine	8.3	2.1	25.9	9.5	27.3	2.8	0.3***	0.3***	0.9
Cytidine monophosphate	17.6	12.2	12.3	9.7	26.9	8.0	0.7	1.4	0.5**
Glutamate	807.3	99.7	442.3	125.3	651.0	43.4	1.2***	1.8***	0.7***
Glycine	3.7	3.2	21.3	34.8	2.4	3.0	1.5	0.2	8.9*
Guanosine	5.5	2.2	4.0	3.5	6.2	1.9	0.9	1.4	0.6*
Histidine	4.2	1.4	3.6	0.5	5.4	1.0	0.8	1.2	0.7**
Isoleucine	3.2	1.4	3.8	1.6	6.1	0.8	0.5***	0.8	0.6**
Isovalerate	3.4	2.2	1.4	0.9	4.1	0.9	0.8	2.5	0.3**
Lactate	19.6	12.2	16.5	6.7	61.4	18.3	0.3***	1.2	0.3***
Leucine	16.8	4.4	16.1	7.0	25.4	5.0	0.7**	1.0	0.6*
Lysine	20.9	8.2	16.3	4.0	25.4	5.3	0.8	1.3	0.6**
NADP^+^	2.2	0.4	3.7	1.6	2.6	0.4	0.8	0.6**	1.4*
Phenylalanine	9.3	1.3	9.0	2.5	13.2	0.8	0.7***	1.0	0.7**
Phosphoenolpyruvic acid	11.2	2.4	18.0	7.0	10.7	5.0	1.1	0.6*	1.7*
sn-Glycero-3-phosphocholine	112.6	24.3	26.2	12.8	31.3	3.3	3.6***	4.3***	0.8
Succinate	18.6	8.2	10.6	2.9	18.5	2.6	1.0	1.8	0.6**
Tyrosine	1.5	0.6	1.3	0.5	2.7	0.7	0.5*	1.1	0.5**
Uracil	9.3	7.6	3.7	3.6	11.1	3.0	0.8	2.5	0.3**
Valine	7.3	0.9	6.0	2.4	10.5	1.4	0.7***	1.2	0.6**
β-Alanine	9.8	4.5	6.3	2.8	11.2	4.1	0.9	1.5	0.6*
2-Hydroxyisobutyrate	2.5	1.6	1.2	0.5	2.0	0.7	1.3	2.0	0.6
3-Hydroxyisovalerate	6.7	4.6	4.7	0.9	4.8	3.5	1.4	1.4	1.0
Adenosine	24.4	10.8	19.0	13.3	29.7	12.0	0.8	1.3	0.6
AMP	5.7	1.7	6.8	5.0	7.5	5.1	0.8	0.8	0.9
Arabinose	17.3	4.5	13.5	7.1	16.4	4.7	1.1	1.3	0.8
Coenzyme A	5.5	1.9	12.1	6.7	11.9	9.1	0.5	0.5	1.0
Formate	94.8	65.7	36.5	21.0	67.2	54.8	1.4	2.6	0.5
Glucose	11.3	9.6	68.0	108.4	2.7	1.1	4.3	0.2	25.6
Glutamine	80.1	55.4	37.6	17.0	62.1	17.7	1.3	2.1	0.6
NAD^+^	15.8	2.2	18.0	3.0	15.3	1.1	1.0	0.9	1.2
Nicotinate	2.1	1.0	1.1	0.4	1.6	0.4	1.3	1.9	0.7
Sucrose	7.0	10.3	84.5	75.6	5.5	7.1	1.3	0.1	15.3
Threonine	11.5	7.1	8.9	5.5	7.7	1.1	1.5	1.3	1.2
Tryptophan	1.4	0.5	2.0	0.6	2.3	1.1	0.6	0.7	0.9
UMP	5.9	3.8	5.2	3.2	8.3	3.4	0.7	1.1	0.6
Uridine	5.7	3.2	5.1	3.3	6.4	4.1	0.9	1.1	0.8

In the first (white background) and second section (grey background) are identified, the metabolites with levels significantly different (p-value ≤ 0.05), at least in one comparison, test, respectively. The fold change and p-value for each comparison in study are shown in the last 3 columns. and the non-differential by non-parametric Wilcoxon. *p-value ≤ 0.05; **p-value ≤ 0.01; ***p-value ≤ 0.001.

**FIGURE 5:**
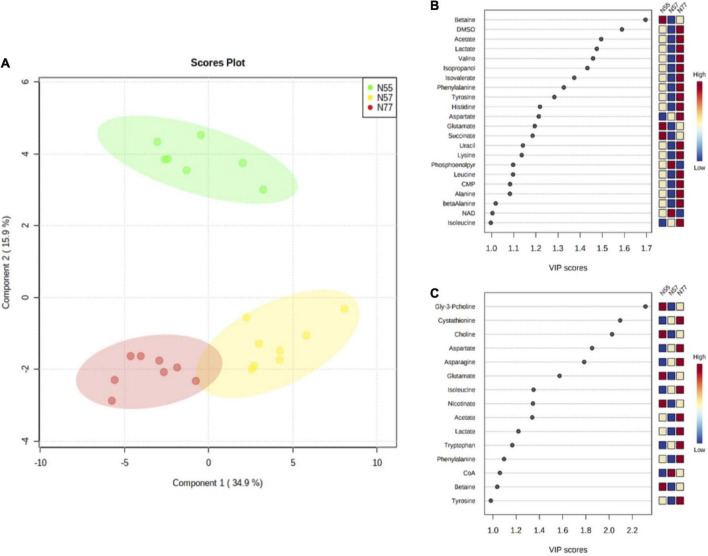
PLS-DA model based on metabolites concentrations for the three pH conditions. **(A)** PLS-DA scores plot of first and second component, showing the discrimination between the 3 experimental conditions, N55 (green) N57 (yellow), and N77 (red) (with 5 components: *R*^2^ = 0.97 and *Q*^2^ = 0.92) using the metabolites levels. Metabolites that most contribute for the separation of the groups based on its VIP scores (>1) for **(B)** the first component and, **(C)** the second component; the colour scale presents the metabolite relative levels among the three experimental conditions.

Metabolite levels were utilised to perform a multivariate analysis. The PLS-DA model discriminates the three conditions (Figure 5) with a *R*^2^ of 0.97 and a *Q*^2^ of 0.92 (the validity of PLS-DA was confirmed by 2000 permutation tests, Supplementary Figure 5). Principal components 1 and 2 explain 49% of the variance among the three experimental conditions. The separation between N77 and N57 occurs in the first component, while the discrimination between N55 and the other conditions occurs in the second component. The metabolites with a VIP score above 1 that contributed more for the discrimination among the three conditions in the first and second component are depicted in Figures 5B,C.

#### Metabolomic analysis at skin vs blood environmental pH

When compared by a univariate analysis, using the non-parametric Wilcoxon test, there were 13 metabolites for which the levels were significantly different between N55 and N77 (Table 1 and Supplementary Figure 6). sn-Glycero-3-phosphocholine, glutamate and choline are increased at the skin pH; while the levels of acetate, asparagine, aspartate, cystathionine, isoleucine, lactate, leucine, phenylalanine, tyrosine and valine are significantly higher on blood pH. Beside the identified compounds, there are resonances from an unidentified NDP-sugar (anomeric resonance at 5.48 ppm) and an unidentified asparagine-like metabolite (resonances at 2.90, 2.98, and 4.00 ppm). The unknown NDP-sugar signals are increased at N55, while the unknown asparagine-like metabolite levels are higher at N77.

**FIGURE 6:**
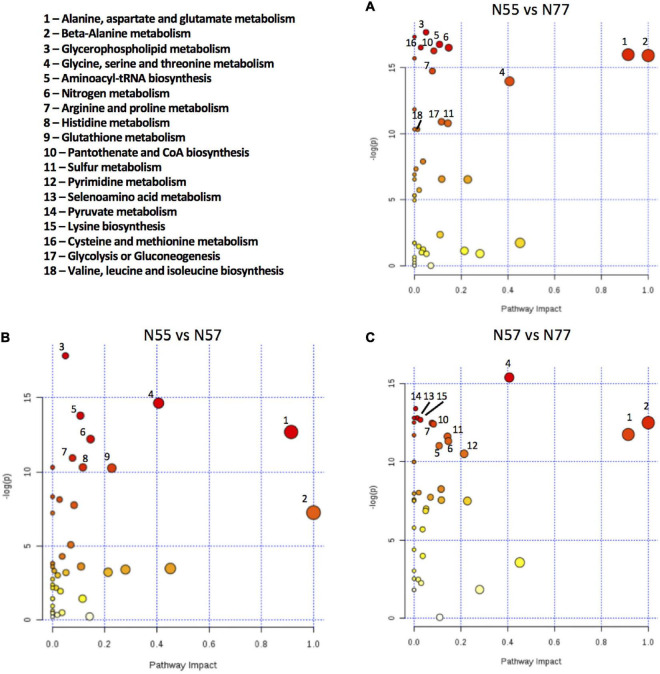
Metabolic pathways altered between the conditions based on metabolite concentrations. Pathway Analysis using the metabolite concentrations, performed in Metaboanalyst, of pairwise comparison among the 3 conditions: **(A)** N55 vs N77, **(B)** N55 vs N57, and **(C)** N57 vs N77; using *Staphylococcus aureus* N315 as a pathway library reference. Pathways named here obey the criteria of negative logarithm of *p*-value higher than 10 (y axis) and a pathway impact > 0, or pathway impact > 0.5 (x axis). Circle size is proportional to the pathway impact value and the colour to the -log(p) value, increasing from white to red.

In order to identify the modulated pathways based on the metabolite concentrations and on the created experimental models we used the Pathway Analysis tool implemented in www.metaboanalyst.ca Comparing N55 vs N77, the most significantly altered pathways are related with amino acid metabolism, namely, alanine, aspartate and glutamate metabolism; β-alanine metabolism; glycine, serine and threonine metabolism; nitrogen metabolism; and arginine and proline metabolism (Figure 6A). These data indicate that amino-acid metabolism is an important process for *S. epidermidis* 19N strain to cope with pH alterations. Also, glycerophospholipid metabolism is altered when comparing *S. epidermidis* 19N grown at skin pH 5.5 with the blood pH condition N77, despite the reduce number of metabolites involved in glycerophospholipid metabolism (2 of 16) that were detect and quantified.

#### Metabolomic comparison of pH shock adaptation vs blood and skin mimicking conditions

Some metabolites with significantly different levels between N55 and N77 conditions are also differential relative to these conditions when *S. epidermidis* 19N cells cope with a quick passage from skin to blood pH (condition N57). Variations were found for additional metabolites between N57 and N55 or N77 (Table 1 and Supplementary Figure 6). Those significantly decreased in N57 when compared with N77 are CMP, β-alanine, uracil, succinate, lysine, isovalerate, guanosine, and histidine. Interestingly, N57 presents the lowest levels of betaine and the highest of phosphoenolpyruvate, NADP^+^ and glycine compared with both N55 and N57. Betaine is an important compatible solute already identified in *S. epidermidis*, which is usually accumulated in stress conditions by several bacteria (Kunin and Rudy, 1991).

The pathway analysis performed for the comparisons N55 vs N57 and N57 vs N77 (Figures 6B,C) suggests that the major pathways influenced by these conditions are the same as the pathways with higher impact in the comparison N55 vs N77, namely alanine, aspartate and glutamate metabolism; β-alanine metabolism and glycine, serine and threonine metabolism.

## Discussion

*Staphylococcus epidermidis* is a commensal bacterial species that inhabits human skin, but when the skin barrier is broken and the immune system is immunocompromised it can cause life-threatening infections, which are difficult to treat (Chessa et al., 2016; Deplano et al., 2016). The most common *S. epidermidis* infections are associated to medical devices. In particular catheters, when introduced through the skin, can give access of skin bacteria to inner regions of the skin and the blood vessels. Catheter-related infections develop through the adhesion of these bacteria to device’s surface followed by the production of biofilms. During the transition from skin to blood, *S. epidermidis* faces multiple environmental changes, but how this bacterium adapts to these changes during infection development and how these contribute to disease is still elusive. One of the factors that constantly changes from skin to the blood and that has been described to be used by bacteria as a signal to remodel bacterial physiology, is the pH. Our hypothesis was that pH change is a key factor for *S. epidermidis* disease development. To test this hypothesis, we analyzed the effect of pH on the metabolism of a commensal *S. epidermidis* strain (19N) through differential proteomics and metabolomics. To mimicking skin we grew *S. epidermidis* at pH 5.5 (N55); to mimic blood we grew *S. epidermidis* at pH 7.4 (N77); and to simulate the beginning of the infectious process, we exposed bacteria to an abrupt pH shift from skin to blood (pH 5.5 → 7.4; N57). The more relevant differential proteins between these three conditions and their metabolic and cellular functions are compiled in Table 2. The results obtained reflect the response of *S. epidermidis* to a change in pH that is similar to the one that it faces when it goes from the skin into the blood. However, the assay conditions are far from mimicking the complex skin and blood environment. As a result, some of the pathways and functions that changed in our experiment might be specific of the growth conditions tested. On the other hand, some of the pH induced changes that occur *in vivo* might have been missed.

**TABLE 2:** Functional analysis of differentially abundant proteins between N55 and N77 conditions.

Increased at pH 5.5		
**Protein**	**ID**	**Gene**

**Glycerolipids pathway**		
Glycerate kinase	SERP0409	garK
Aldehyde dehydrogenase	SERP1729/2084	aldA
Glycerol dehydrogenase	SERP2346	gldA
Dihydroxyacetone kinase	SERP2344/5	
Triacylglycerol lipase	SERP2388	geh-1
Processive diacylglycerol beta-glucosyltransferase	SERP0606	ypfP
**Oxidative phosphorylation/Oxidative stress**		
Lactaldehyde dehydrogenase	SERP2080	
Alcohol dehydrogenase, zinc-containing	SERP1785/6	
Pyruvate oxidase	SERP2115	
Catalase	SERP0903	katA
Glutathione peroxidase	SERP0872	gpxA-1
**Phosphate metabolism**		
Polyphosphate kinase	SERP2047	ppk
Exopolyphosphatase	SERP2046	
**Betaine Biosynthesis**		
Oxygen-dependent choline dehydrogenase	SERP2176	betA
Betaine aldehyde dehydrogenase	SERP2177	betB
**Folate Biosynthesis**		
2-Amino-4-hydroxy-6-hydroxymethyldihydropteridine pyrophosphokinase	SERP0155	folK
Dihydropteroate synthase	SERP0153	folP
GTP cyclohydrolase 1		folE
**Glutamate Metabolism**		
Glutamate synthase	SERP0109	gltB
Formimidoylglutamase	SERP1919	hutG
**Cell Division**		
N-acetylmuramoyl-L-alanine amidase	SERP2263	
Essential cell division protein	SERP0745	ftsL
Two-component system WalK/WalR, regulator of histidine kinase	SERP2531	YycI
ClpXP adapter protein	SERP0581	SpxH
**Peptidoglycan synthesis**		
Aminoacyltransferase FemA		femA
**Drug Transport**		
Drug resistance transporter	SERP1944	
Drug transporter	SERP1945	

**Increased at pH 7.4**		
**Protein**	**ID**	**Gene**

**Purine/Nucleotide Metabolism**		
Xanthine phosphoribosyltransferase	SERP0067	xpt
Adenylate kinase	SERP1810	adk
N5-carboxyaminoimidazole ribonucleotide synthase	SERP0650	purK
Phosphoribosylformylglycinamidine synthase subunit PurL	SERP0654	purL
Phosphoribosylformylglycinamidine cyclo-ligase	SERP0656	purM
Phosphoribosylaminoimidazole-succinocarboxamide synthase	SERP0651	purC
Phosphoribosylamine-glycine ligase	SERP0659	purD
Phosphoribosylformylglycinamidine synthase	SERP0653	purQ
Amidophosphoribosyltransferase	SERP0655	purF
**Glycolysis/Pyruvate metabolism**		
Glyceraldehyde-3-phosphate dehydrogenase	SERP1250	gapA-2
Fructose-bisphosphate aldolase	SERP1732	fbaA
Pyruvate, phosphate dikinase	SERP1129	ppdK
Pyruvate, water dikinase	SERP2169	
Phosphoenolpyruvate carboxykinase	SERP1353	pckA
Alcohol dehydrogenase	SERP0257	adh
Acetate kinase	SERP1275	ackA
Pyruvate formate lyase activating enzyme	SERP2365	pflA
Formate acetyltransferase	SERP2366	pflB
Dihydrolipoyl dehydrogenase	SERP2327	
Dihydrolipoamide acetyltransferase	SERP2324	
**Oxidative Phosphorylation**		
Cytochrome aa3-600 menaquinol oxidase subunit ii	SERP0646	qoxB
Cytochrome aa3-600 menaquinol oxidase subunit iii	SERP0644	qoxC
Succinate dehydrogenase / fumarate reductase, cytochrome b subunit	SERP0730	sdhC
NADH:flavin oxidoreductase/fumarate reductase, flavoprotein subunit	SERP2381	
**Butanoate metabolism**		
Acetoin(diacetyl) reductase	SERP2257	budC
Diacetyl reductase [(S)-acetoin forming]	SERP2379	butA
Acetoin dehydrogenase	SERP2325	acoA
Acetoin dehydrogenase	SERP2326	acoB
Acetyl-CoA C-acetyltransferase	SERP0220	vraB
**Arginine/Proline metabolism**		
Pyrroline-5-carboxylate reductase	SERP1065	proC
Proline dehydrogenase	SERP1324	putA
Nitric oxide synthase oxygenase	SERP1451	
Ornithine carbamoyltransferase;	SERP2351	arcB-2
Carbamate kinase	SERP2352	arcC
Delta-1-pyrroline-5-carboxylate dehydrogenase	SERP2128	rocA
**Phosphoenolpyruvate:carbohydrate phosphotransferase system (PTS)**		
Fructose-specific IIABC components	SERP2260	
Sucrose-specific IIBC components	SERP1900/68	
Glucose-specific EIICBA component	SERP2114	ptsG
6-Phospho-beta-galactosidase	SERP1789	lacG
Tagatose 1,6-diphosphate aldolase	SERP1792	lacD
**Membrane Transporters: Hemin, Iron and Mn**		
Multidrug resistance efflux pump	SERP1767	SepA
Flotillin-like protein	SERP1140	FloA
RND family efflux transporter	SERP0014	
ABC transporter, substrate-binding protein	SERP0290	ArsC1
Transferrin receptor	SERP0949/403	
Iron compound ABC transporter, ATP-binding protein	SERP0402	
Hemin import ATP-binding protein	SERP0015	HrtA
Hemin transport system permease protein	SERP0016	HrtB
Iron transporter	SERP1139	
Siderophore synthetase	SERP1779	
Staphyloferrin B biosynthesis	SERP1781	
**Extracellular Proteases/Cell Adhesion**		
Glutamyl endopeptidase		SspA
Staphopain A Extracellular cysteine protease		sspB
SdrG protein	SERP0207	SdrG
**Regulators of virulence factors expression**		
Transcriptional regulator, AraC family	SERP1972	
Two Component Signal Transduction System PhoPR, Sensory box histidine kinase	SERP1255	phoR
Two Component Signal Transduction System SaeRS, Response regulator	SERP0365	saeR
Staphylococcal Accessory Regulator family	SERP1849/76/79	SarR/Z/V
**t-RNA modification**		
NADPH-dependent 7-cyano-7-deazaguanine reductase	SERP0394	queF
Queuine trna-ribosyltransferase	SERP1203	tgt
Epoxyqueuosine reductase QueH	SERP2147	queH
tRNA (guanine-N(1)-)-methyltransferase	SERP0806	trmD

### *S. epidermidis* metabolic and biological activities induced at skin pH

#### Promotion of bacterial growth

We found that *S. epidermidis* 19N at the skin pH had a shorter lag phase, a faster growth rate and reached a higher biomass level when entering the stationary phase (see Supplementary Figure 1) than at blood pH. These results suggest that this strain has a higher fitness at pH 5.5. This is not surprising, given the commensal nature of the 19N strain, and is in accordance with previous data, wherein *S. epidermidis* strains from B clonal lineage were shown to have higher growth rates when exposed to acidic pH (Espadinha et al., 2019). However, our results contrast with those obtained by Iyer et al. who observed that another *S. epidermidis* strain belonging to the A/C cluster (*S. epidermidis* ATCC12228) was insensitive to increasing pH values (pH 5–7) (Iyer et al., 2021). This discrepancy may be explained by the fact that the assays were performed with an A/C cluster strain, with distinct growth conditions: in that case the growth was performed in a microtiter plate.

**FIGURE 7:**
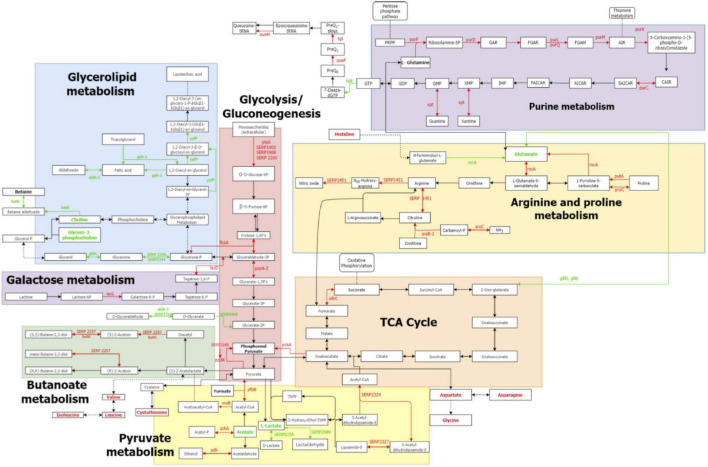
Metabolic pathways altered between N55 vs N77 based on the proteomic and metabolomic data. Reconstruction of the principal metabolic pathways that changed in *S. epidermidis* 19N when growth at blood pH (N77) when compared with skin pH (N55). Names of metabolites and enzymes increased in N77 are in red, while those increased in N55 are in green. Metabolites determined in the study but that do not show significant differences are in black, while metabolites undetected are in grey. Full arrows represent reactions, dashed arrows represent connections with other pathways or hidden reactions.

Differential analysis of the *S. epidermidis* proteome and metabolome identified significant variations in the metabolic and cellular processes between N55 and N77 conditions, suggesting that this pH shift is an important signal for *S. epidermidis* metabolic reprogramming (Figure 7).

The increased growth rate at pH 5.5 observed in this study was corroborated by the proteomic data obtained, in which several proteins involved in peptidoglycan synthesis, cell division and DNA replication were overrepresented in N55 conditions. This is the case of a homologue of FemA, an important enzyme for the synthesis of peptidoglycan pentaglycine bridge (Hegde and Shrader, 2001); N-acetylmuramoyl-L-alanine amidase (Sle1) (Fold Change = 22), FtsL, Yycl (Takada and Yoshikawa, 2018) and SpxH (Awad et al., 2019), involved in cell division; and SERP2046/SERP2047 and FolK/FolP which are associated to the biosynthesis of triphosphate nucleotides (Bowlin and Gray, 2021) and folate, two factors relevant for DNA synthesis.

#### Cellular membrane composition remodelling

A metabolic pathway that was activated at the skin pH was that associated to the metabolism of phospholipids. This is a vital facet of bacterial physiology that begins with the synthesis of the fatty acids by fatty acid synthases and ends up in cellular membrane biogenesis (Zhang and Rock, 2008). Membranes are formed by glycerophospholipids, composed of two fatty acids, a glycerol moiety, a phosphate group and a variable head group. Attached to the cell membrane of Gram-positive bacteria are lipoteichoic acids (LTA), an alditolphosphate-containing polymer that is linked via a lipid anchor to the membrane. It has been previously described that bacteria change the composition of fatty acids or the head groups in the glycerophospholipids in response to several abiotic stresses, modifying the membrane proprieties, like fluidity and charge (Willdigg and Helmann, 2021). Moreover, membrane-linked LTA, can suffer different modifications, including the addition of d-alanyl and glycosyl residues in *S. aureus*, which can alter their charge, adhesion proprieties and interaction with human host immunity (van Dalen et al., 2020).

When the 19N strain was grown at acidic pH we observed that the levels of enzymes and metabolites involved in glycerolipids and fatty acids metabolism and in lipoteichoic acids biosynthesis were increased. Among these were enzymes responsible for the synthesis of glycerophospholipids precursors, (AldA, GarK, GldA and SERP2344/5). Additionally, we observed the accumulation of choline and glycero-3-phosphocholine, building blocks of glycerolipids. Similarly, we observed that enzymes catalysing the removal of fatty acids from di- and triacylglycerols (Geh-1 and YpfP) were accumulated at the acidic pH, suggesting the occurrence of fatty-acid and LTA composition remodelling in this condition. This is in accordance with a previous report in which *Streptococcus mutans* strains increased the levels of monounsaturated and longer-chain fatty acids in its membrane to increase membrane fluidity when exposed to acidic pH (Quivey et al., 2000). On the other hand, changes in lipoteichoic acids amount and composition may have implication in both cell division via regulation of autolytic cell wall enzymes (muramidases) and in protection against cationic antimicrobial peptides (Percy and Gründling, 2014), which are part of the innate immunity in human skin. Overall, our results indicate that *S. epidermidis* respond to acidic stress by increasing the biosynthesis of cell membranes, probably as a result of an increased growth and cell division at this pH, and by remodelling the cellular membrane composition.

#### Induction of protection against other skin abiotic stresses

Skin is a harsh environment for bacteria to live in. In the skin bacteria will have to cope with multiple abiotic stresses like acidic pH, osmotic stress promoted by the high salt concentration, oxidative stress induced by UV exposure, mechanical and chemical stresses by external factors, the presence of antimicrobial peptides and nutrient limitation.

We found that *S. epidermidis* response to skin acidic stress (pH 5.5) included the induction of pathways associated to response to other abiotic stresses of the skin, like osmotic and oxidative stress. This was the case of the production of choline, a compatible solute (Kunin and Rudy, 1991; Graham and Wilkinson, 1992) described to be associated to resistance to osmotic stress (Kunin and Rudy, 1991; Graham and Wilkinson, 1992; Peddie et al., 1998). Besides, this metabolite is also a precursor of other compatible solutes like betaine. In our study, we observed that betaine levels were very similar in the two pH conditions tested, however, the two dehydrogenases (BetA and BetB) involved in betaine synthesis from choline were incremented in the skin pH. Similarly, we observed that at the skin pH, proteins involved in protection against oxidative stress (KatA and GpxA-1) were also induced.

Our results suggest that the low pH of the skin is a signal for the production of metabolites and proteins that protect against other skin abiotic stresses like osmotic and oxidative stress.

#### Accumulation of glutamate

When grown in condition N55, *S. epidermidis* showed the accumulation of glutamate and higher levels of glutamate synthase (GltB and GltD) and formimidoylglutamase (HutG) (Kocianova et al., 2005). These enzymes are involved in the synthesis of glutamate from 2-oxoglutarate and N-formimino-L-glutamate, respectively, linking the increase in glutamate with TCA cycle and histidine degradation. Simultaneously the level of the enzyme responsible for the introduction of glutamate into the transformation of proline to ornithine (RocA) is also diminished, contributing to the described glutamate increase at N55. This regulation of the urea cycle allows to reduce the consumption of NH_3_, making it more available to increase the levels of ammonium ion contributing to intracellular pH increase. Glutamate is a key metabolite in *Staphylococcus* metabolism, being pivotal in the connection between different metabolic pathways. In particular, glutamate was shown to be an important factor for *S. aureus* survival under acidic conditions (Yee et al., 2019). Moreover L-glutamate is the precursor of poly-γ-glutamate (PGA), a L-glutamate and D-glutamate polymer, that was found to confer resistance to the host innate immunity, namely to antimicrobial peptides, and to protect *S. epidermidis* from high salt concentrations, key features of its natural environment - the human skin (Kocianova et al., 2005).

#### Membrane transporters

Membrane transporters profile is also altered by the growth at the two different pHs. At skin pH, the levels of two multidrug transporters, SERP1944 and SERP1945, are increased. The function of these transporters is unknown, but it was observed that in the presence of dimeric isoquinoline, an antibiotic, SERP1944 transcripts are increased in *S. epidermidis* (Cecil et al., 2011). Also, a transporter involved in the copper export, CopA, is increased in this condition. CopA is responsible for the copper export in diverse *Staphylococcus* species, protecting the organism from the copper antimicrobial effects and being important for copper homeostasis (Kaur et al., 2022).

#### Generation of chemical energy

The main generators of reducing power at the skin pH were most probably lactaldehyde dehydrogenase (SERP2080) and a zinc-containing alcohol dehydrogenase (SERP1785/6) that were accumulated in this condition. The mechanism were they are involved may generate a higher extrusion of intracellular protons (Korem et al., 2010), and is in accordance with the increment of pyruvate oxidase (SERP2115) involved in menaquinone biosynthesis, also associated with a more active oxidative phosphorylation.

### *Staphylococcus epidermidis* metabolic and biologic activities induced at blood pH

#### Production of chemical energy

One of the most significant changes observed at blood pH was in the central metabolism, including the increase of enzymes involved in glycolysis, pyruvate metabolism and oxidative phosphorylation. These pathways constitute the main means of cellular production of redox potential and ATP that are the major sources of energy for bacteria.

In particular, at blood pH, we observed an increase in the abundance of the phosphoenolpyruvate-dependent sugar phosphotransferase system (PTS)— a major carbohydrate active-transport system that transfers the phosphoryl group of phosphoenolpyruvate to incoming sugar substrates concomitantly with their translocation across the cell membrane. Four proteins from this system (PtsG, SERP1900, SERP1968 and SERP2260) specific for glucose, sucrose and fructose transport, and enzymes generating phosphoenolpyruvate from oxaloacetate (PckA) and pyruvate (PpdK and its regulator SERP2169) support the fuelling of glycolysis in the N77 condition. This is in accordance with the increment at N77 of two proteins from the lactose catabolic process via tagatose-6-phosphate (LacD and LacG). However, the only two metabolites directly involved in glycolysis and TCA cycle that were possible to quantify in our metabolomic analysis, namely phosphoenolpyruvate and succinate, were not significantly different between skin and blood pH conditions.

Other proteins that were increased in N77 condition were several membrane dehydrogenases (GapA-2, SdhC, SERP2324/7, SERP2327, QoxB, and QoxC), suggested by Uribe-Alvarez et al. (Uribe-Alvarez et al., 2016) to be electron donors to menaquinone, the quinone known to be utilised for respiration in *S. epidermidis*.

In addition to glycolysis, pyruvate metabolism (see Figure 7) was also more active at blood pH, with several enzymes (AckA, Adh, PflB, PflA, and VraB) more abundant at N77. Moreover, the production of acetate and lactate, by-products, or final products of the pyruvate metabolism, were also increased at N77, indicating that in this condition the cells, although in aerobiosis, are also using fermentation to generate energy. Actually, in blood, oxygen is less available to bacteria than in the skin. Thus turning on the fermentative pathway might be an adaptive response that allows *S. epidermidis* to adapt better to blood/infection environment. Additionally, *S. aureus* growing in biofilms, considered a micro-aerophilic environment, also accumulate these acid fermentation products. To counteract the consequent drop in cytoplasmic pH driven by these acids, *S. aureus* is described to induce the activation of butanoate pathway (Zhu et al., 2007)—a system that regulates the NAD/NADH ratio and promotes carbon storage (Vasu et al., 2015). In our study we observed a similar response, wherein proteins involved in butanoate pathway (ButA, SERP2257, SERP2325 and SERP2326) were also accumulated at blood pH when compared to skin pH.

Overall, our results suggest that there is a higher amount of available cellular energy at pH 7.4 than at pH 5.5. Our hypothesis is that *S. epidermidis* is adapted and optimised to live on the skin, wherein energetic requirements are set to the minimum, whereas exposure to blood pH is an accidental stress that to be resolved requires extra energy levels.

#### Activation of nucleotide metabolism

Another metabolic pathway that was incremented at blood pH conditions was the synthesis of nucleotides (Figure 7). Nucleotide metabolism plays a crucial function in bacterial physiology, producing the nucleic acids needed for DNA replication and RNA transcription. In addition, nucleobases also constitute the molecular basis of cellular energy molecules such as ATP and NADH, and many coenzymes are derived from nucleobase monomers. The triggering of the nucleotide metabolism at blood pH is thus in accordance with the observed increase of proteins involved in cellular energy production, described above. Also is illustrative of an active cellular metabolism.

Particularly we observed an increase of proteins within pathways associated to the synthesis of purines. These included five of the seven enzymes that convert L-glutamine into the purine 1-(5-Phospho-D-ribosyl)-5-amino-4-imidazolecarboxylate (CAIR), and xanthine phosphoribosyltransferase (Xpt) that catalyzes the conversion of the purine xanthine to its monophosphate form—a metabolite that can then be reused for the synthesis of several RNA forms involved in protein biosynthesis. However, the quantified metabolites participating in these pathways, like glutamine, adenine, guanosine and adenosine, were not found differentially accumulated in none of the pH conditions tested. The increase in purine metabolism was previously described to be crucial for growth in blood and for the infection process of different bacteria like *S. aureus* (Connolly et al., 2017; Li et al., 2018), *Escherichia coli*, *Salmonella enterica*, and *Bacillus anthracis* (Samant et al., 2008). On the other hand, it was also reported in *Bacillus subtilis* that one of the consequences of intracellular acidification is the loss of purines and pyrimidines from DNA at a greater rate than at neutral pH (Lindahl and Nyberg, 1972), what might explain the low levels of enzymes involved in purine synthesis in the acidic skin pH.

Our data indicate that exposure of the *S. epidermidis* commensal to blood pH triggered a similar change toward a more active and pathogenic-like purine metabolism.

#### Induction of tRNA modification

We additionally observed that exposure of *S. epidermidis* to blood pH induced the pathways associated to the modification of tRNA. tRNAs are key factors in protein synthesis, establishing the link between the messenger RNA (mRNA) and the amino acids chain that make up a protein. tRNA modifications constitute an extra layer of protein synthesis regulation, being implicated in the accuracy and efficiency of decoding, on inhibition and induction of toxins and proteins of the translation apparatus, and in the monitoring of tRNA integrity and stability. Moreover, their roles during adaptation to environmental stresses and to infection have started to be acknowledged. Recent examples of tRNA modifications regulating host-pathogen interactions include the regulation of immune responses, antibiotic resistance, expression of virulence genes, and bacterial persistence (Antoine et al., 2021). The most modified region of tRNA is the anticodon stem loop (ASL). In particular tRNA modifications in this region have been implicated in the expansion and restriction of the decoding properties of a given tRNA.

In our study we found that the growth at a higher pH induced the biosynthesis of enzymes involved in tRNA modifications occurring in the ASL region, namely those associated to the replacement of a guanosine by a queuosine (QueF, QueH and Tgt) and to guanine methylation (TmrD). These modifications have been described to be implicated in virulence and response to environmental conditions in different bacterial species. An example of this interaction was observed in *Shigella flexneri*, wherein a link was established between the reduction of Tgt homolog activity and a decrease in pathogenicity (Durand et al., 2000). Moreover, the ratio between queuosine and its precursor levels, was shown to be regulated by O_2_ availability in *E. coli* and *Salmonella enterica*, which led to the proposal that tRNA modification can be a marker of cellular oxygen requirements (Edwards et al., 2020). On the other hand, the methylation of tRNA guanine 37 promoted by TmrD was found to be essential in diverse pathogenic bacteria, like *Pseudomonas aeruginosa*, *S. enterica*, *Streptococcus pneumoniae* and *E. coli* because it suppresses translational frameshift errors at proline codons (Edwards et al., 2020).

Our results suggest that *S. epidermidis* growth at pH 7.4 induced tRNA modifications that are essential to guarantee the fidelity of protein synthesis, but that simultaneously can turn on the pathogenicity pathways and prepare *S. epidermidis* to the low-oxygen environment of the blood.

#### Activation of amino acids synthesis and catabolism

Amino acids, besides being the building blocks of protein synthesis, are important for sustaining cell integrity and metabolic homeostasis, and are precursors in the synthesis of other molecules (Halsey et al., 2017; Alreshidi et al., 2019). Also they have been described to have a role in cell signalling, stress protection, energy production and host-pathogen interactions (Christgen and Becker, 2019).

In condition N77, we observed that many amino acids such as leucine, isoleucine, valine, glycine, aspartate, asparagine, phenylalanine, and tyrosine were present in a higher concentration than in N55 condition, suggesting the existence of an active metabolism involving these amino acids at the higher pH. These results are in accordance with previous data obtained for *S. aureus*, for which the total amount of free amino acid levels was described to be higher at pH 7 when compared to a lower pH (Alreshidi et al., 2016; Murphy et al., 2018). These amino acids might be fuelling the synthesis of new proteins to adapt to higher pH. Additionally, they might be being used as carbon sources for energy production.

Actually, we found that a higher amount of the enzymes involved in proline synthesis (ProC) and catabolism (PutA and RocA) was produced at pH 7.4, when compared to pH 5.5 (Figure 7; Halsey et al., 2017). Proline catabolism was recently demonstrated by Fey and coworkers to be important for *S. aureus* growth, particularly during infection. This is believed to be due to the fact that glucose can become limited in an infectious situation, and proline can function as an alternative source of carbon and energy to the TCA cycle. In *S. epidermidis* infections, the proline-rich host matrix protein collagen is one of the first to cover the surface of the catheters and prosthesis and the induction of the collagen catabolism may be used as a survival strategy in the low nutrient environment of the infection.

#### Induction of virulence factors

Because it is mainly a commensal bacteria, *S. epidermidis* does not contain many virulence factors, when compared to other more pathogenic staphylococcal species like *S. aureus*. The majority of virulence factors are associated to adhesion, biofilm formation and evasion of human immune system. In this study when we grew *S. epidermidis* in pH 7.4 we observed that many systems regulating virulence in *S. epidermidis* as well as specific adhesins were promoted when compared to pH 5.5 condition.

This was the case of SaeR and PhoR, which are part, respectively of SaeRS and PhoPR Two-Component Systems. These systems are modular transduction pathways that allow bacteria to adapt to environmental conditions by inducing the biosynthesis of a wide array of cell surface and secreted virulence factors (Burgui et al., 2018). SaeRS is related with the regulation of staphylococcal immune evasion proteins, adhesins and γ-haemolysin (Handke et al., 2008) and PhoPR involved in phosphate scavenging/transport under phosphate-limiting conditions and in wall teichoic acids metabolism (Prunty, 2017). Also, levels of DNA-binding transcription factors belonging to the Staphylococcal Accessory Regulator (Sar) family (SarR, SarV, and SarZ) were increased in N77. Members of this family are important in the regulation of the *Staphylococcus* virulence, being key players in different processes like colonisation, biofilm production, autolysis and haemolysis (Manna et al., 2004; Wang et al., 2008; Ballal and Manna, 2009; Rowe et al., 2011). Additionally, a Rbf homologue (SERP1972) was found more abundant at N77. Previous studies in *S. epidermidis* have demonstrated that this transcription regulator from the AraC family, can directly downregulate sarR and sarX and induce the production of the *ica* operon, responsible for the production of the polysaccharide intercellular adhesion (PIA), one of the main components of *S. epidermidis* biofilm (Rowe et al., 2016; Espadinha et al., 2019). However, 19N strain does not have the *ica* operon and was shown not to produce biofilm at pH 7.4 (Espadinha et al., 2019), suggesting that in this case Rbf function should be different.

Another type of proteins that were also increased in the blood pH were the extracellular proteases (SspA and SspB) that contribute to the colonisation and infection of human tissues (Rice et al., 2001) and the cell surface protein SdrG that mediates adhesion of *S. epidermidis* to fibrinogen (Vanzieleghem et al., 2015), a host matrix protein that coats the medical device once is introduced into inner human tissues.

We also observed the production of a higher amount of nitric oxide synthase oxygenase (SERP1451) at pH7.4 when compared to pH5.5. In *S. aureus*, nitric oxide synthase was previously described to contribute to virulence (Gaupp et al., 2012; Kinkel et al., 2016), resistance to oxidative stress (Mogen et al., 2017) and resistance to antibiotics (Chaffin et al., 2012), all features that are important during an infectious process, to invade tissues, evade immune system and circumvent treatment.

Condition N77 also led to increased levels of flotillin-homolog protein FloA. This protein has been shown in *S. aureus* to be a scaffold protein involved in the binding of proteins and facilitate physical interaction of multi-enzyme complexes located at the membrane level such as the type VII secretion system (T7SS) (Koch et al., 2017) and membrane-associated RNase Rny, which forms part of the RNA-degradation machinery called the degradosome (Mielich-Süss et al., 2017). Both systems can be implicated in virulence. While T7SS are described to be involved in the export of virulence effectors to host cells (Tran et al., 2021), the degradosome is known to regulate the amount of targetted sRNA transcripts that negatively control *S. aureus* toxin biosynthesis (Koch et al., 2017).

Our results indicate that the growth at the blood pH served as a signal for the biosynthesis of several virulence factors implicated in the adhesion to the host cells, invasion of host tissue, and evasion of host immunity.

#### Induction of multidrug and iron transporters

Additionally we found that multidrug resistance efflux pumps like SepA and SERP0014 (Resistance-Nodulation-Cell division, RND, family efflux transporter) were increased at blood pH, when compared to skin pH. In *S. aureus*, SepA was shown to be involved in resistance to antiseptics, like acriflavine (Narui et al., 2002) and members of the RND transporter super-family are known to catalyse the active efflux of many antibiotics and chemotherapeutic agents (H + /drug antiporters) (Lekshmi et al., 2018). The accumulation of these efflux pumps in the infection scenario might allow *S. epidermidis* to survive disinfection, usually performed before the introduction of the medical device, and antibiotic treatment. This can constitute the opportunity of *S. epidermidis* to enter inner layers of the skin, resist antibiotics and cause infection.

Moreover, membrane proteins related with transport of iron were also accumulated at the blood pH. Iron is an essential nutrient for most bacterial species because it is a co-factor of many essential metabolic enzymes. Human tissues have usually a low concentration of free iron, but this element is abundant in the host in the form of the metalloporphyrin haem (the cofactor of haemoglobin and myoglobin) and the glycoproteins transferrin and lactoferrin. Due to the low availability of free Fe, there is continuous arms race for iron between bacterial pathogens and hosts. Thus as a survival strategy in the context of infection, bacteria have evolved several mechanisms of iron acquisition, including the removal of haem-iron from host hemoproteins, the acquisition of transferrin and lactoferrin-bound iron, and the uptake of free inorganic iron (Oliveira et al., 2021).

Haem from haemoglobin is usually transported into the bacterial cell via ABC transporters. However it is also toxic to bacteria because it generates reactive oxygen species that damage lipids, proteins and nucleic acids. For that reason bacteria have developed haem detoxification systems that enable bacteria to export these toxic compounds from bacterial cells. The acquisition of free iron and iron from transferrin and lactoferrin can be done via the binding to bacterial receptors or through the production by bacteria of siderophores, potent iron chelators that are afterward transported into cells. Despite being a major cause of bloodstream infections, there is a lack of information on iron acquisition systems by *S. epidermidis*. While no siderophore has been described in *S. epidermidis* so far, there are findings suggesting that this species is able to produce at least one siderophore (Oliveira et al., 2017).

In our study we found that growth of *S. epidermidis* at blood pH induced the biosynthesis of proteins with homology to iron compound ABC transporters (SERP0403 SERP1139), transferrin receptors (SERP0402, SERP0949) and siderophore synthases (SERP1779, SERP1781) as well as the haem detoxification system (HrtA/B), suggesting that pH 7.4 could be a signal for *S. epidermidis* to switch on the iron scavenging machinery. These results are in agreement with a previous work with *S. saprophyticus* for which an increment of iron acquisition enzymes was also noticed when these bacteria were grown in alkaline conditions (Souza et al., 2019).

### *Staphylococcus epidermidis* metabolic and biologic activities induced by an abrupt skin-to-blood pH change

During the infection process that involves the transfer of *S. epidermidis* from skin to blood, the bacterial cells need to deal with a rapid pH increase of 2 units. To understand which are the metabolic processes associated to this rapid pH shift we grew the 19N strain first at pH 5.5 and afterward at pH 7.4 (condition N57). We observed that in this condition 19N strain grew faster than at pH5.5 or 7.4. These results indicate that, in N57 condition, pH homeostasis mechanisms probably were able to maintain the optimal intracellular pH, in such a way that favoured 19N growth. This increase in growth when bacterial cells are subjected to a sudden pH increase might contribute to a successful infectious process.

Proteomic and metabolomic analysis showed a clear discrimination of the protein and metabolite levels between N57 and the two conditions not involving pH changes (N55 and N77) (Figures 2D, 3). The number of proteins with differential abundances between the conditions mimicking cells adapted to skin pH (N55) and blood pH (N77) is 243. Much less differential proteins were determined for the comparison of both conditions with N57 (145 and 88, respectively) (Figure 2D). Among these, there were some metabolic mechanisms that are probably related with the quick response to pH increase.

One of the mechanisms that appeared to be affected when bacteria were subjected to a sudden pH change was the betaine biosynthesis pathway that was downregulated in condition N57, when compared to N55 and N77, as illustrated by the lower abundance of choline dehydrogenase (BetA) and betaine-aldehyde dehydrogenase (BetB), together with betaine transporters, BetL and SERP0246. Although betaine has been mainly assigned to a osmoprotection function, other roles have been described for this metabolite, like in pH homeostasis (Bay and Turner, 2012; Wargo, 2013) and as a global metabolic regulator (Figueroa-Soto and Valenzuela-Soto, 2018).

Additionally, both glutamate and succinate levels were reduced in condition N57 when compared to N55 and N77, while NADP^+^ and phosphoenolpyruvate were incremented. These three metabolites are involved in redox cell homeostasis, which could be imbalanced by the sudden pH increase. This hypothesis is corroborated by the fact that both aldo-keto reductase (SERP0244) and multicopper oxidase (mco), two proteins involved in redox reactions were differentially abundant between the conditions N55 vs N57 and N57 vs N77 and not between N55 vs N77.

Other proteins whose levels varied only in condition N57 included proteins involved in sulphur metabolism (O-acetylserine dependent cystathionine beta-synthase (SERP0094) and adenylyl-sulphate kinase), aspartate metabolism [2,3,4,5-tetrahydropyridine-2,6-dicarboxylate N-acetyltransferase (dapD)] and cell wall synthesis [accessory Sec system protein Asp1 (SERP2279)].

## Conclusion

The drastic changes observed on the proteome and metabolome of 19N bacterial cells at blood pH when compared with those at skin pH suggest that this pH change is an important signal for remodelling *S. epidermidis* 19N metabolism toward an infection context. At the skin pH the main processes involved promotion of bacterial growth, adjustment of membrane fluidity/charge, pH regulation, redox cell homeostasis, protection against osmotic stress and glutamate accumulation. On the other hand, exposure to the higher circulating blood pH induced an increase of *S. epidermidis* strain metabolic activity, of nucleotide metabolism, tRNA modification, amino acid synthesis and catabolism, and stimulated iron acquisition, drug resistance and bacterial virulence. These results indicate that at skin pH *S. epidermidis* seems to be more prepared to deal with environmental factors, while at blood pH is tuned to circumvent aggressions from the host.

## Data availability statement

The datasets presented in this study can be found in online repositories. The names of the repository/repositories and accession number(s) can be found below: ProteomeXchange Consortium via the PRIDE partner repository with the dataset identifier PXD034825 and doi: 10.6019/PXD034825. Metabolomic data is available at the NIH Common Fund’s National Metabolomics Data Repository (NMDR) website, the Metabolomics Workbench, https://www.metabolomicsworkbench.org where it has been assigned Project ID ST003341.

## Author contributions

SS and LPG cultured the isolates and performed the proteomic and metabolomic experiments and helped in data analysis. LGG perfomed the metabolomic and carried out the data analysis and interpretation and helped in the writing of the manuscript. JA provided the proteomic data, carried out data analysis, and revised the manuscript. AC planned the research work, peformed data analysis and interpretation, and wrote the manuscript. MM planned the research work, helped in the interpretation of data, and wrote the manuscript. All authors read and approved the final manuscript.

## Footnotes

1
https://string-db.org

2
https://www.metabolomicsworkbench.org

3
www.metaboanalyst.ca
