# Supplementary material for: Skin-to-blood pH shift triggers metabolome and proteome global remodelling in Staphylococcus epidermidis
Source: Front Microbiol. 2022 Sep 28;13:1000737. doi: 10.3389/fmicb.2022.1000737 (PMC9554481; doi:10.3389/fmicb.2022.1000737)
Supplement: Supplementary file 2 [file Image_1.pdf]

A

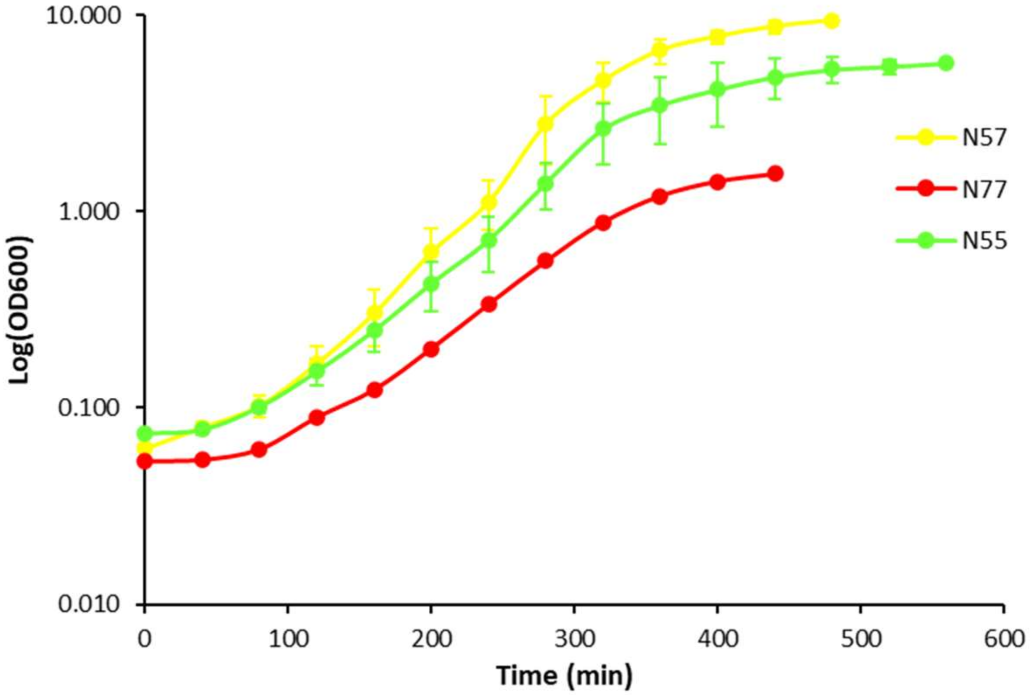

B

	Growth Rate			Generation Time			p-value for comparison of growth rate/generation time	
**Condition**	Mean (h^{-1})	SD (h^{-1})	CV (%)	Mean (h)	SD (h)	CV (%)	**N77**	**N57**
**N55**	0.817	0.024	2.927	0.848	0.025	3.006	3.19E-04	1.92E-04
**N57**	0.978	0.020	2.065	0.709	0.016	2.231	1.08E-06	-
**N77**	0.705	0.012	1.701	0.984	0.018	1.815	-	-

Supplementary Figure 1: Growth curves of *Staphylococcus,epidermidis* commensal strain 19N. (A) Each dot represents the average of several independent assays for condition N55 in green (n=4), N57 in pink (n=4) and N77 in blue (n=1). Bars represent standard deviations. **(B)** Calculated growth parameters. The p-values determined by the Student t-test for the comparison of growth rate/generation time for each pair of experimental conditions. (SD-standard deviation; CV-coefficient of variation.)

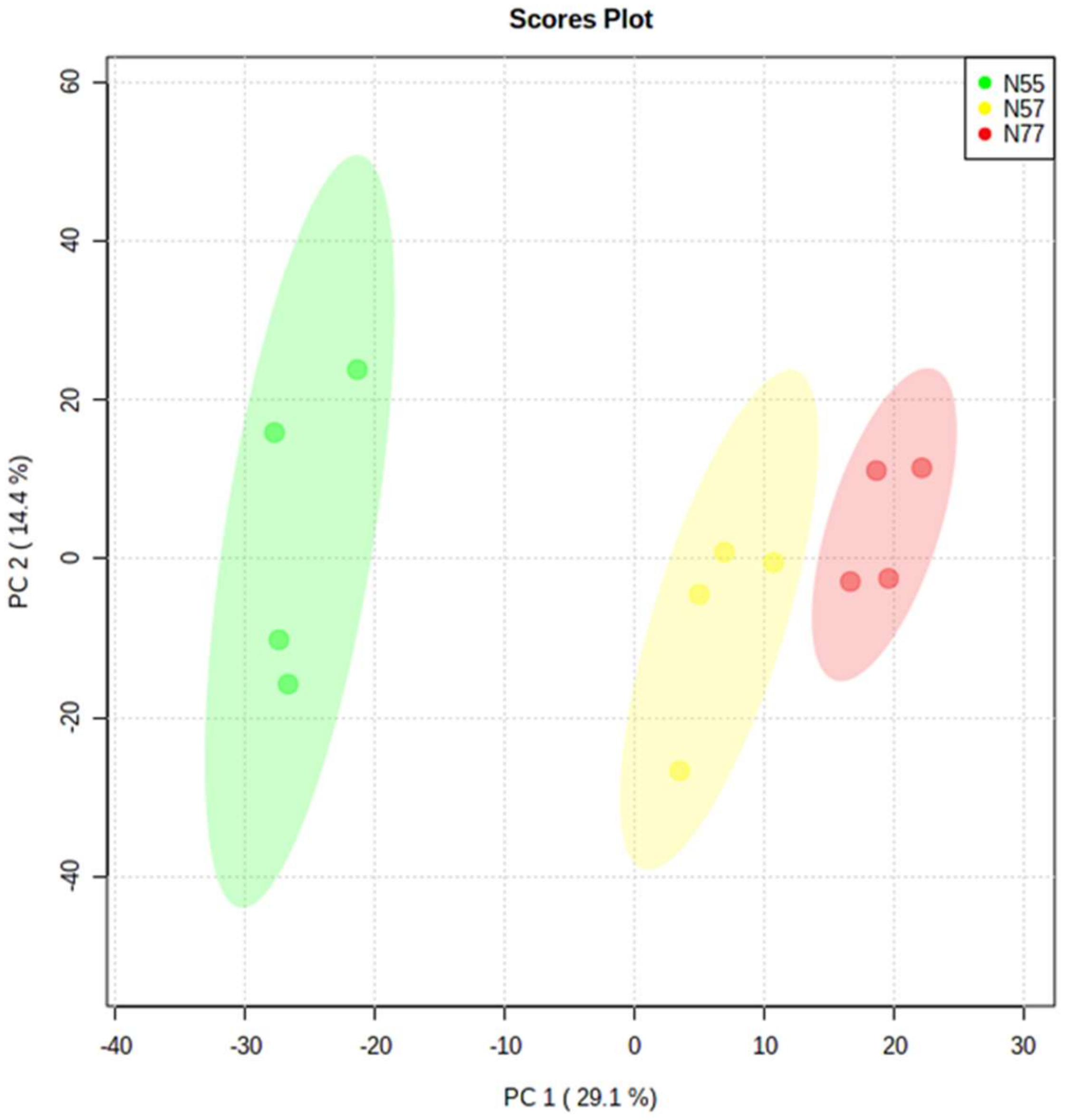

Supplementary Figure 2: PCA model based on proteins levels among different conditions. PCA scores plot for the first and second components, showing the discrimination among the 3 experimental conditions, N55 (green), N57 (yellow), and N77 (red). Areas in green, yellow and red represent the 95% confidence region. Dots within each area represent the replicates for each condition (n=4).

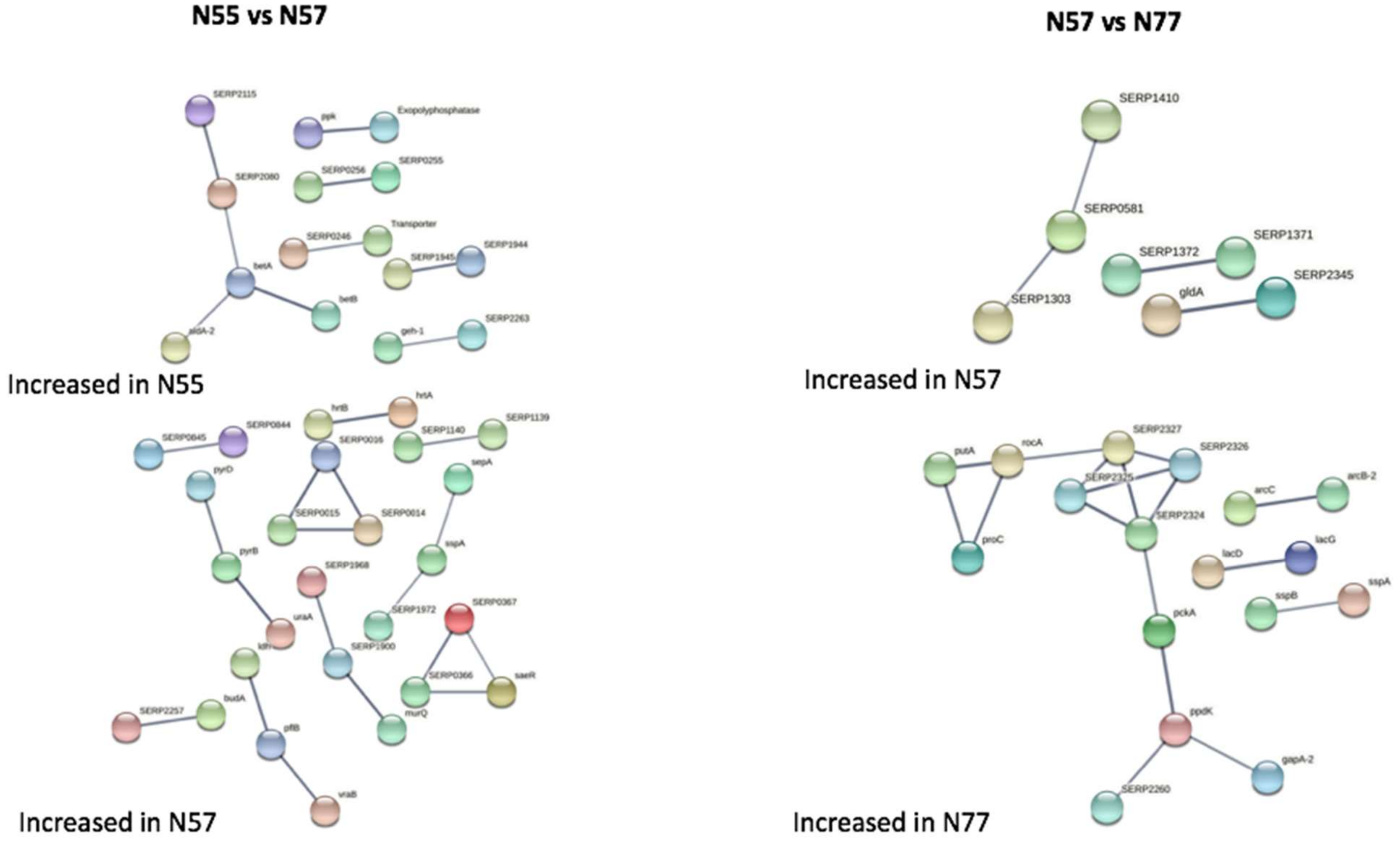

Supplementary Figure 3: STRING network of differentially abundant proteins between N55 vs N57 and N57 vs N77.

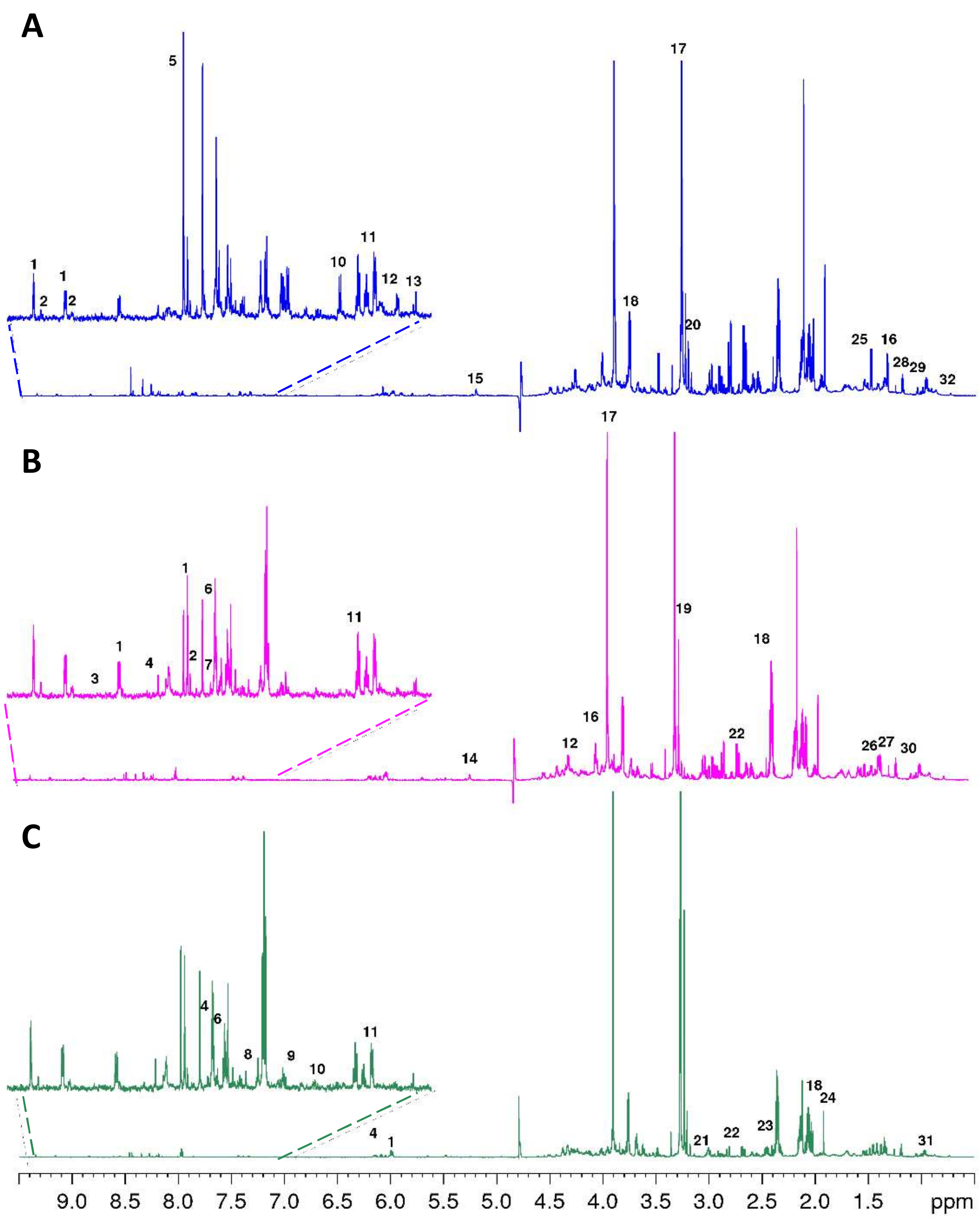

Supplementary Figure 4. Representation of typical 1D ^{1}H NMR spectra for each experimental condition. (A) N77, **(B)** N57 and **(C)** N55. Numbers correspond to the identified metabolites: 1- NAD^{+}, 2-$NADP^{+}$, 3- nicotinate, 4- AMP, 5- formate, 6- adenosine, 7- adenine, 8- guanosine, 9- tryptophan, 10- uracil, 11- phenylalanine, 12- tyrosine, 13- histidine, 14- sucrose, 15- glucose, 16- lactate, 17- betaine, 18- glutamate, 19- sn-glycero-3-phosphocholine, 20- choline, 21- lysine, 22- aspartate, 23- succinate, 24- acetate, 25- alanine, 26- 3-hydroxyisovalerate, 27- ethanol, 28- valine, 29- isoleucine, 30- leucine, 31 – isovalerate and 32 - coenzyme A.

Diapositivo 4

1 Colocar A, B e C para cada figura
Já está incluído na legenda
Ana Coelho; 26/01/2022

A

B

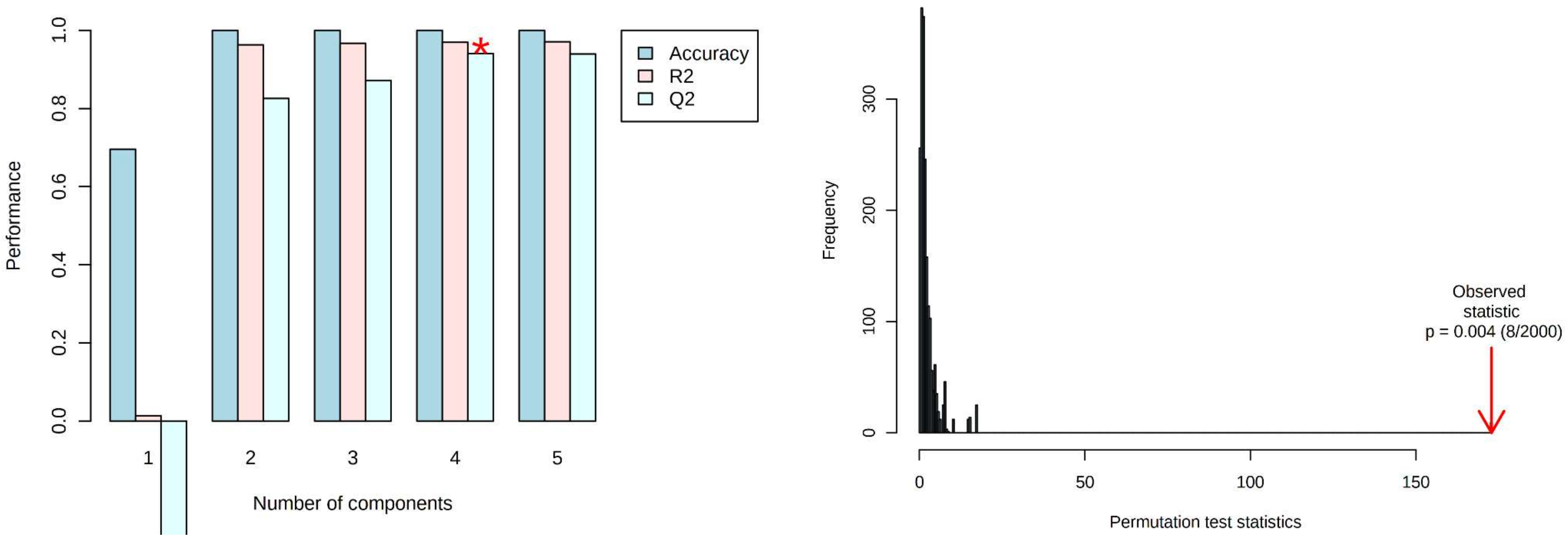

Supplementary Figure 5. Validation parameters of PLS-DA model for metabolomics data. (A) R2, Q2 and accuracy for the different components of the PLS-DA model. It was the selected the 3 components model. **(B)** The permutation test (n=1000)shows that the model is statistically valid (p-value = 0.004).

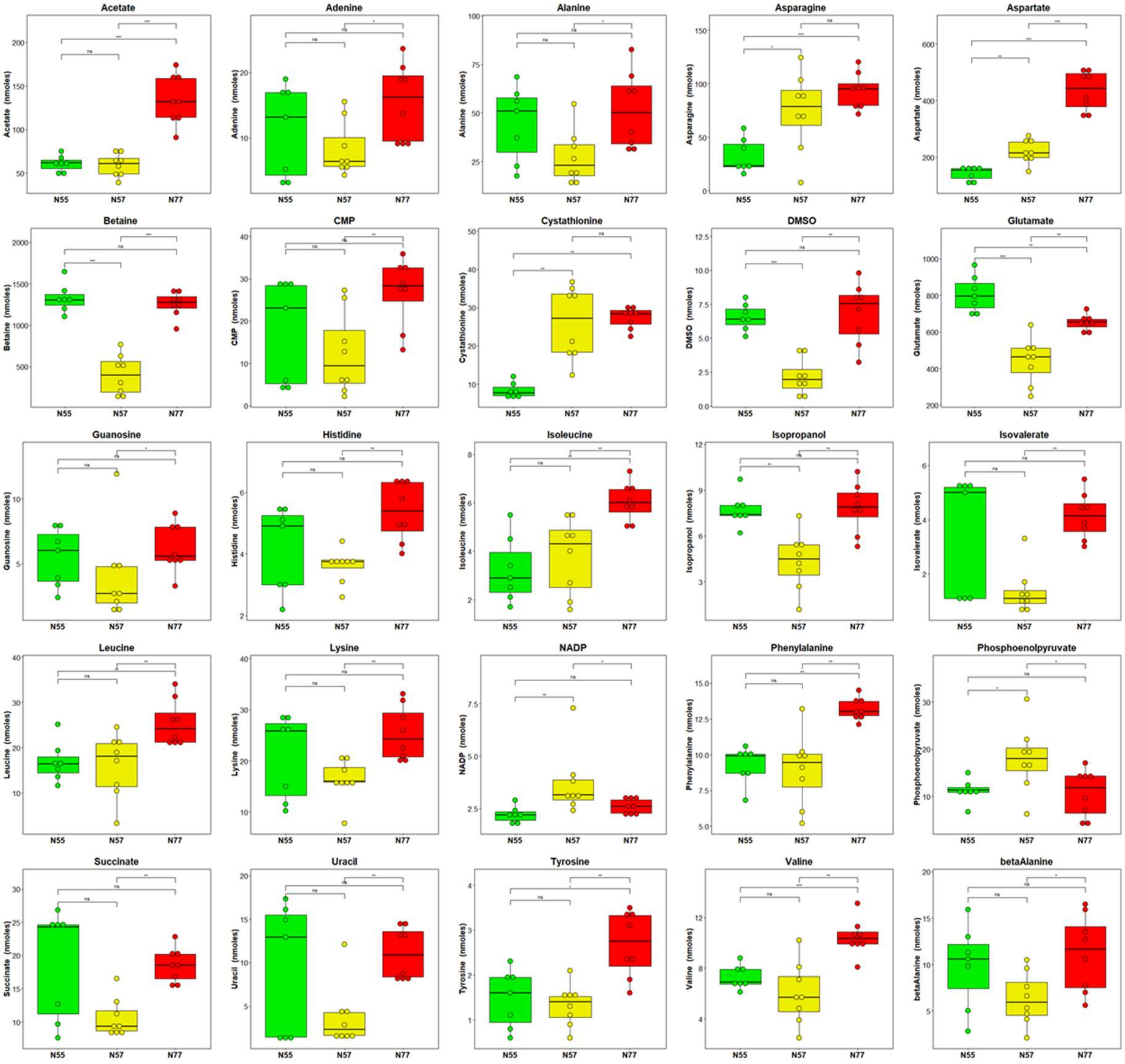

Supplementary Figure 6. Box plot representation for the differential metabolites determined by the Wilcoxon test.

Supplementary Figure 6. Box plot representation for the differential metabolites determined by the Wilcoxon test.

The three conditions N55, N57 and N77 are represented in green, yellow and red. In the upper section of each box plot are shown the p-values for the comparisons among each pair of experimental conditions: ns–no significant, p-value lower than *0.05, ** 0.01 or *** 0.001.
